# Myoblasts and macrophages are required for therapeutic morpholino antisense oligonucleotide delivery to dystrophic muscle

**DOI:** 10.1038/s41467-017-00924-7

**Published:** 2017-10-16

**Authors:** James S. Novak, Marshall W. Hogarth, Jessica F. Boehler, Marie Nearing, Maria C. Vila, Raul Heredia, Alyson A. Fiorillo, Aiping Zhang, Yetrib Hathout, Eric P. Hoffman, Jyoti K. Jaiswal, Kanneboyina Nagaraju, Sebahattin Cirak, Terence A. Partridge

**Affiliations:** 1 Center for Genetic Medicine Research, Children’s Research Institute, Children’s National Health System, Washington, DC 20010 USA; 2 Institute for Biomedical Sciences, The George Washington University School of Medicine and Health Sciences, Washington, DC 20052 USA; 3 Department of Pediatrics, The George Washington University School of Medicine and Health Sciences, Washington, DC 20052 USA; 4 Department of Pharmaceutical Sciences, School of Pharmacy and Pharmaceutical Sciences, Binghamton University, Binghamton, NY 13902 USA; 5 Institute for Human Genetics, University Hospital Cologne, Cologne, 50923 Germany; 6 Department of Pediatrics, University Hospital Cologne, Cologne, 50923 Germany; 7 Center for Molecular Medicine, University of Cologne, Cologne, 50931 Germany

**Keywords:** Drug delivery, Macrophages, Neuromuscular disease, Skeletal muscle

## Abstract

Exon skipping is a promising therapeutic strategy for Duchenne muscular dystrophy (DMD), employing morpholino antisense oligonucleotides (PMO-AO) to exclude disruptive exons from the mutant *DMD* transcript and elicit production of truncated dystrophin protein. Clinical trials for PMO show variable and sporadic dystrophin rescue. Here, we show that robust PMO uptake and efficient production of dystrophin following PMO administration coincide with areas of myofiber regeneration and inflammation. PMO localization is sustained in inflammatory foci where it enters macrophages, actively differentiating myoblasts and newly forming myotubes. We conclude that efficient PMO delivery into muscle requires two concomitant events: first, accumulation and retention of PMO within inflammatory foci associated with dystrophic lesions, and second, fusion of PMO-loaded myoblasts into repairing myofibers. Identification of these factors accounts for the variability in clinical trials and suggests strategies to improve this therapeutic approach to DMD.

## Abstract (web summary)

Exon skipping is a strategy for the treatment of Duchenne muscular dystrophy, but has variable efficacy. Here, the authors show that dystrophin restoration occurs preferentially in areas of myofiber regeneration, where antisense oligonucleotides are stored in macrophages and delivered to myoblasts and newly formed myotubes

## Introduction

Duchenne muscular dystrophy (DMD) is a severe and relentlessly progressive myopathy that results from out-of-frame or nonsense mutations in the X-linked *DMD* gene that disrupt the mRNA open reading frame and prevent translation of dystrophin protein^1, 2^. In DMD patients, the lack of dystrophin leads to persistent muscle degeneration, inflammation, and fibrosis, with associated loss of myofiber structural integrity and functional strength^3^. The milder, allelic variant, Becker muscular dystrophy (BMD) results from in-frame mutations in the *DMD* gene that permit expression of an internally truncated, partially functional dystrophin isoform^4, 5^. This understanding has inspired a therapeutic strategy in which antisense oligonucleotides (AO) are designed to restore the open reading frame by excluding disruptive exons so as to permit translation of a truncated dystrophin protein^6–9^, thus converting DMD to a milder BMD phenotype^4, 5^. Preclinical testing of AOs favors the phosphorodiamidate morpholino oligomers (morpholino, PMO) on the basis both of efficacy of exon skipping and dystrophin restoration and of low toxicity^10–19^. Despite its promise for DMD, the path through clinical trials to FDA approval has been complicated by the technical challenges of accurate measurement of the variable and patchy distribution of restored dystrophin in the context of the ethical limitations on dystrophic muscle biopsies as clinical endpoint measures^20–24^.

Our recent investigations found no evidence of association between exon skipping efficacy and residual PMO concentrations within muscles, or the preference of systematically delivered PMO for particular muscle groups or fiber types, suggesting the existence of other constraints^25^. Following IV injection, PMO remained detectible in the serum for less than 2 h (half-life ~30 min), while the majority accumulated in the liver and kidneys or was excreted in the urine^26–28^. Meanwhile, trace amounts of PMO were found distributed throughout the muscle, where it diminished slowly throughout the first week. The resulting pharmacokinetic picture is of rapid elimination of even high concentrations of PMO from the bloodstream, and limited entry into muscle fibers that, together, severely restrict its availability to myonuclei where it exerts its biological activity^26–28^.

To elucidate the factors that influence the efficacy of exon skipping in preclinical studies and clinical trials, we have traced the route of PMO delivery into nuclei of regenerating muscle in the dystrophic *mdx* mouse model. To identify the “birthdate” of myogenic cells in regenerating muscle lesions in *mdx* mice, we used 3 day phases of bromodeoxyuridine (BrdU) administration staggered relative to a single intravenous dose of PMO. This defined a narrow “window of opportunity” in which PMO is able to efficiently penetrate differentiating myoblasts and actively repairing myofibers to yield productive exon skipping and dystrophin expression. These newly identified pathophysiological features trigger a fundamental revision of the pharmacokinetics underlying the distribution of PMO-induced exon skipping for DMD and highlight new targets for improvement of this therapeutic strategy.

## Results

### Myofiber “birthdating” strategy to investigate PMO delivery

**Fig. 1:**
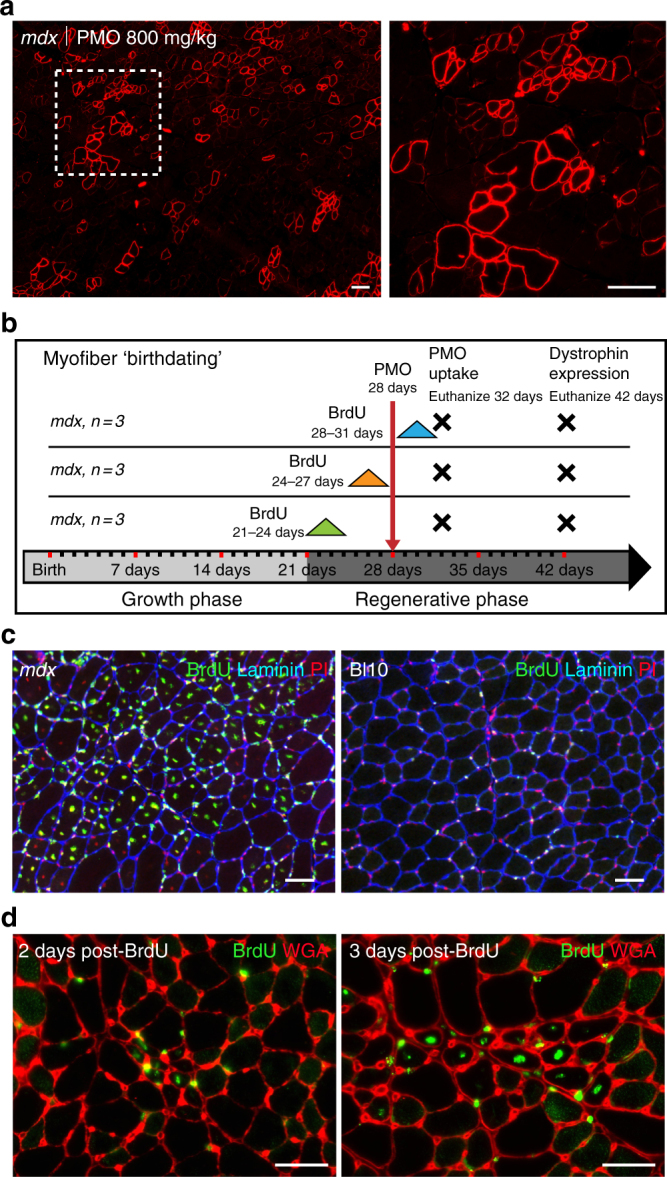
PMO and BrdU co-administration to investigate PMO uptake and efficacy relative to regeneration. **a** Patchy dystrophin expression shown in *mdx* quadriceps 2 weeks after single IV dose of PMO (800 mg/kg, *n* = 3). *Dashed box* shows magnified inlay, *scale bars* represent 100 μm. **b** Schematic representation of parallel PMO-BrdU labeling assays to investigate relationship between PMO localization, dystrophin expression, and muscle regeneration. **c** Onset of *mdx* pathology at 4 weeks characterized by widespread regeneration shown by BrdU labeling of centrally nucleated fibers. Bl10 muscle without regeneration shown as control with only minor labeling of interstitial nuclei. BrdU (*green*), laminin (*blue*), and propidium iodide (PI, *red*). Scale bars represent 50 μm. **d** BrdU labeling of newly regenerated fibers shown in 4 week *mdx* gastrocnemius 48 and 72 h following a 24 h feeding of BrdU. *Scale bars* represent 50 μm

Our investigation was guided by two earlier observations: (1) a general correlation of PMO entry with muscle fiber damage^29^ and (2) patchy distribution of dystrophin expression produced by systemic delivery of PMO that echoes the pattern of DMD/*mdx* muscle lesions^25, 30^ (Fig. 1a). We chose to simplify interpretation of our experiments by concentrating on the first round of spontaneous lesions that appear in muscles of the *mdx* mouse beginning at ~21 days of age^31–33^. A single, high intravenous dose of PMO was administered on postnatal day 28 and BrdU was provided ad libitum in the drinking water over the 3 day periods from 21 to 24 days, 24 to 27 days, or 28 to 31 days of age (Fig. 1b).

We first confirmed a previous observation that BrdU administration in wild-type mice of this same age, labeled only sporadic cells lying between muscle fibers but not myonuclei^34^ (Fig. 1c). In contrast, age-matched *mdx* mice exhibited conspicuous scattered patches of labeling throughout the muscle; these comprised both BrdU-labeled myonuclei, indicating newly regenerated myofibers, and interstitial cells, largely denoting proliferating inflammatory cells (Fig. 1c). Observations over the first few days of BrdU administration revealed the first BrdU-labeled centrally positioned myonuclei 2 days post administration, establishing this as the minimum transit time from S-phase through myoblast differentiation and fusion (Fig. 1d). By day 3, most lesions are solidly populated by BrdU-labeled myonuclei, indicating that the staggered 3 day BrdU protocol is appropriate to analyze regenerative cohorts of myogenic cells.

### PMO-induced dystrophin expression specific to dystrophic lesions

To identify the “birthdates” of myoblasts that gave rise to dystrophin^+ve^ fibers in vivo, BrdU was provided for staggered 72 h intervals over 21–24, 24–27, and 28–31 days of age, with respect to a single systemic dose of PMO targeting *Dmd* exon 23 (800 mg/kg; 5′-GGCCAAACCTCGGCTTACCTGAAAT-3′^12, 35, 36^) administered on day 28 (Fig. 1b). Mice were euthanized 14 days after PMO delivery to assess dystrophin expression in the triceps, gastrocnemius, and quadriceps, typically high-responding muscle groups^25^ (Fig. 1b). This pinpoints the stages of myoblast proliferation and fusion at which PMO was most effectively delivered into the muscle fibers within sites of spontaneous degeneration/regeneration.

**Fig. 2:**
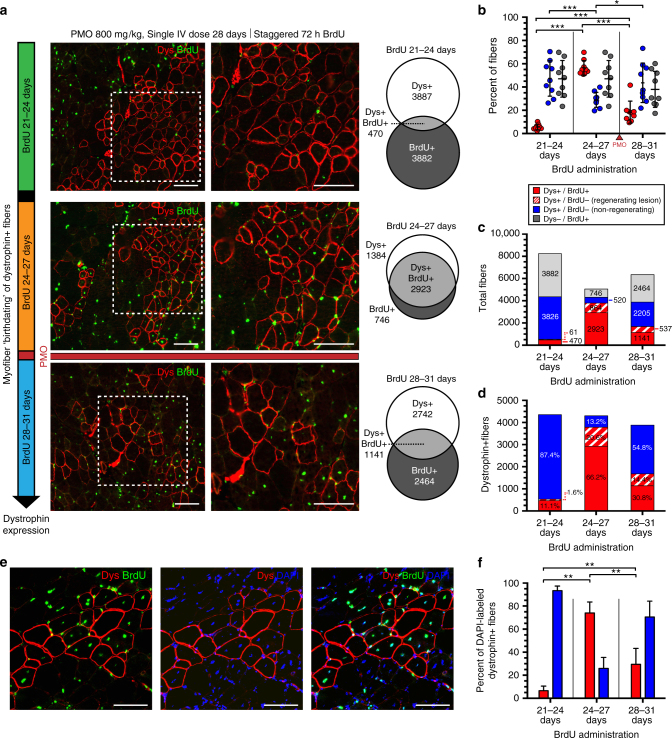
Systemic PMO delivery targets dystrophin expression to spontaneously regenerating *mdx* myofibers. **a** Dystrophin expression in 6 week *mdx* mice (i.e., tricep, gastrocnemius, and quadriceps) after single IV dose of PMO (800 mg/kg, *n* = 3). PMO injected at day 28 together with staggered pulses of BrdU (i.e., 21–24 days, 24–27 days, 28–31 days). Dystrophin (Dys, red) and BrdU^+ve^ myonuclei (*green*) 14 days after PMO. Venn diagrams demonstrate the overlap between dystrophin expression (Dys^+ve^) and myofiber regeneration (BrdU^+ve^). **b** Quantification of Dys^+ve^/BrdU^+ve^, Dys^+ve^/BrdU^−ve^, and Dys^−ve^/BrdU^+ve^ myofibers for each BrdU cohort shown as a percentage of labeled fibers. Data represented as scatter plot with SD; *n* = 3 *mdx* mice per BrdU cohort. **c** Quantification of total fibers characterized as Dys^+ve^/BrdU^+ve^ (*red*), Dys^+ve^/BrdU^−ve^ within a regenerating lesion (*dashed red*), Dys^+ve^/BrdU^−ve^ isolated revertants (*blue*), or Dys^−ve^/BrdU^+ve^ (*gray*). **d** Quantification of BrdU co-localization in dystrophin^+ve^ fibers; Dys^+ve^/BrdU^+ve^ (*red*), Dys^+ve^/BrdU^−ve^ within a regenerating lesion (*dashed red*), Dys^+ve^/BrdU^−ve^ (*blue*) (*n* = 3). **e** BrdU-DAPI co-staining procedure to confirm BrdU specificity and assess prevalence of central myonuclei per myofiber within a given cross-section. **f** Quantification of BrdU-labeling for centrally nucleated (DAPI^+ve^), dystrophin^+ve^ myofibers. *Scale bars* represent 100 μm. Statistical analysis performed by Mann–Whitney nonparametric test; ***p < 0.001, **p < 0.01, *p < 0.05

We quantified all myofibers that labeled for dystrophin, BrdU or both. In all mice, BrdU labeling produced sporadic patches of central BrdU^+ve^ nuclei within small-diameter myofibers (Fig. 1c, d). Myofibers derived from myogenic precursor cells that had proliferated during the 3 days prior to PMO delivery showed markedly greater incidence of dystrophin expression than those of the other BrdU cohorts. In the BrdU 24–27 days cohort, some 57% of fibers were double-positive for dystrophin and BrdU (Fig. 2a, b), while 66% of all dystrophin^+ve^ fibers contained at least one BrdU^+ve^ nucleus and 87% were localized within actively regenerating foci as indicated by a predominance of BrdU-positive myonuclei (Fig. 2c, d). By contrast, in mice given BrdU during days 21–24, only 11% of dystrophin^+ve^ fibers contained a BrdU-labeled central nucleus (Fig. 2a–d). Interestingly, BrdU administration after PMO treatment, during days 28–31, produced co-labeling in 31% of labeled myofibers (Fig. 2a–d), indicating some myoblast proliferation subsequent to PMO administration. This tail end of our defined “window” of optimal PMO uptake perhaps indicates persistence of a local reservoir of PMO after its elimination from the blood. Absolute numbers of BrdU-labeled fibers or dystrophin-expressing fibers did not differ between these three dosing cohorts, indicating a constant rate of regeneration and of PMO uptake over this time (Supplementary Fig. 1a).

The absence of a BrdU^+ve^ myonucleus within some dystrophin^+ve^ fibers, reflects, in part, the fact that within 8 µm cross-sections many myofiber profiles do not contain a nucleus, thus underestimating the proportion of fibers that do contain a BrdU^+ve^ myonucleus. In confirmation, ~90% of dystrophin^+ve^ fibers contained at least one BrdU-labeled central nucleus within four adjacent serial sections along the axis of the fiber (Supplementary Fig. 1b). We later found that high concentrations of 4′,6-diamidino-2-phenylindole (DAPI) would mark BrdU^−ve^ nuclei and confirmed that ~25% of myofiber profiles in any given cross-section did not include a nucleus (Supplementary Fig. 1c). Assessment of dystrophin^+ve^ fibers in BrdU/DAPI-labeled muscles (Fig. 2e, f) echoed our previous analysis, identifying the same optimal 3–4 day window prior to PMO delivery.

In summary, efficient uptake of PMO on day 28 was minimal in cells that had proliferated over the 21–24 day BrdU exposure period, but occurred in 75% of those that proliferated between 24 and 27 days, and in 30% of those which had undertaken at least one S-phase after exposure to PMO (Fig. 2f). These results imply that PMO enters muscle fibers predominantly during the final phases of regeneration.

### Mechanisms of PMO entry persist in older *mdx* mice

**Fig. 3:**
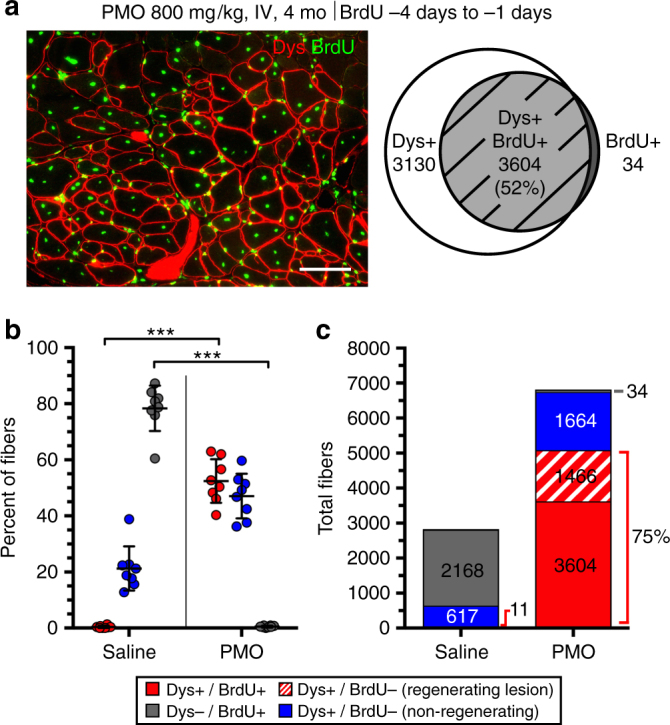
PMO entry of regenerating myofibers in mature *mdx* mice. **a** Dystrophin expression specific to BrdU^+ve^ regenerated myofibers in aged *mdx* muscle (triceps, gastrocnemius, and quadriceps) following a single IV dose of PMO (800 mg/kg, *n* = 3) at 13 weeks of age in conjunction with BrdU administered for 72 h prior to PMO delivery. Dystrophin expression (Dys, *red*) and BrdU^+ve^ central myonuclei (*green*) 14 day after PMO to evaluate dystrophin restoration relative to muscle regeneration. Venn diagram demonstrates the overlap between dystrophin expression (Dys^+ve^) and myofiber regeneration (BrdU^+ve^). *Scale bar* represents 100 μm. **b** Quantification of Dys^+ve^/BrdU^+ve^, Dys^+ve^/BrdU^−ve^, and Dys^−ve^/BrdU^+ve^ myofibers shown as a percentage of labeled fibers. Data represented as scatter plot with SD. **c** Quantification of labeled fibers classified as Dys^+ve^/BrdU^+ve^ (*red*), Dys^+ve^/BrdU^−ve^ within a regenerating lesion (*dashed red*), Dys^+ve^/BrdU^−ve^ (*blue*), or Dys^−ve^/BrdU^+ve^ (*gray*) (*n* = 4). Statistical analysis performed by Mann–Whitney nonparametric test; ***p < 0.001

To determine whether this mechanism is limited to the initial onset period of conspicuous pathology, we treated mature 13-week-old *mdx* mice with high-dose PMO, following a 3-day administration of BrdU. Fewer actively regenerating lesions and dystrophin-expressing fibers were observed in these older mice, and therefore we were easily able to count all fibers within a muscle cross-section that were dystrophin^+ve^ and/or contained a BrdU^+ve^ myonucleus. In PMO-treated mice, these counts showed a strong bias toward dystrophin/BrdU co-labeling, where in fibers marked by either label, ~52% were double-labeled (Fig. 3a–c), while 99% of all BrdU^+ve^ fibers were dystrophin^+ve^ (Fig. 3b). Thus, almost all fibers that had undergone regeneration during the 3 days prior to PMO delivery had gone on to produce dystrophin. This tighter association between exon skipping and active regeneration may be attributable to the lower frequency of pathological episodes in older *mdx* mice, lowering the incidence of BrdU labeling in separate, temporally overlapping lesions.

Small isolated pockets of dystrophin^+ve^/BrdU^−ve^ fibers were noted in both saline and PMO-treated groups (Fig. 3c). We interpret these as expanded clusters of “revertant” fibers typical in the *mdx* mouse at this age^37, 38^. In saline-treated mice <1% of these “revertant” fibers were BrdU^+ve^, indicating a low incidence of active expansion as predicted in earlier studies^37, 38^.

### Inflammatory exudation targets PMO to sites of regeneration

More direct observations of PMO entry into muscle were conducted with fluorescein-conjugated PMO (F-PMO), identified by immunostaining for the fluorescein tag. After a single intravenous dose, F-PMO was predominantly detected in muscle regions exhibiting active regeneration associated with dense patches of mononucleated interstitial inflammatory cells (Supplementary Fig. 2a–d). Functional exon skipping activity of F-PMO was confirmed through reverse transcriptase quantitative PCR (RT-qPCR) and dystrophin immunostaining following systemic delivery (Supplementary Fig. 3a–d). Importantly, the slight negative charge caused by conjugation with carboxyfluorescein had no significant effect on exon skipping efficacy.

To confirm PMO accumulation within inflammatory/regenerating lesions as a key factor modulating its uptake, we assessed intramuscular PMO and exon skipping in wild-type Bl10 mice injected intravenously with 400 mg/kg of F-PMO and observed only sparse interstitial distribution of the F-PMO throughout the muscle, likely corresponding to resident macrophages. However, no F-PMO was observed within myonuclei of muscle fibers (Supplementary Fig. 4a). RT-qPCR revealed ~1% exon skipping, modestly higher than the <0.1% detected in saline-treated Bl10 or *mdx* muscles, but significantly below the >10% in muscles of PMO-treated *mdx mice* (Supplementary Fig. 4b, c).

**Fig. 4:**
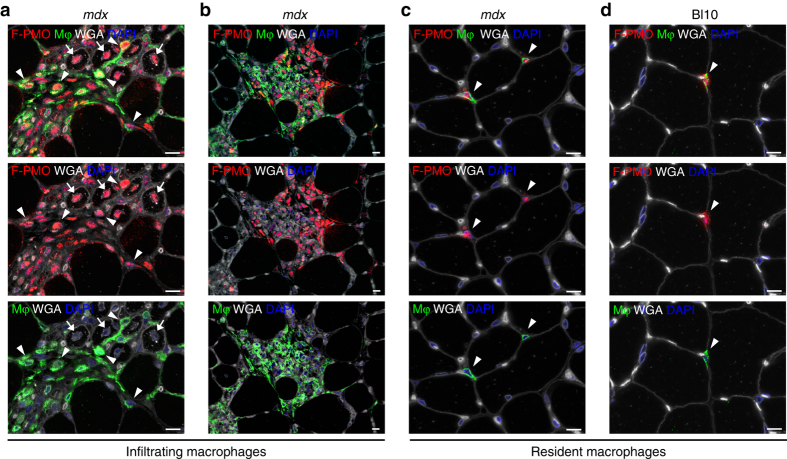
PMO penetrates infiltrating inflammatory cells targeting regions of active muscle regeneration. **a** PMO uptake within infiltrating macrophages, targeting areas of regeneration after systemic F-PMO delivery in 4 week *mdx* mice (400 mg/kg, *n* = 3); PMO uptake observed within myonuclei is marked by *arrow*, while PMO-loaded macrophages (Mφ) are marked by *arrowheads* and immunostained with F4/80. **b** Widespread PMO accumulation within a large population of infiltrating macrophages and other inflammatory cells targeting degenerating myofibers. **c**, **d** Sporadic PMO uptake within interstitial, resident macrophages localized in non-regenerating *mdx* (**c**) and wild-type (**d**) muscle. *Arrowheads* mark examples of PMO^+ve^ macrophages. Muscles were analyzed following single injection of F-PMO, mice were euthanized 4 days post-PMO delivery. PMO, *red*; macrophage F4/80, *green*; DAPI, *blue*; WGA, *white*; *scale bars* represent 10 μm

Staining for the macrophage marker F4/80 revealed that a majority of PMO^+ve^ interstitial cells within muscle lesions correspond to infiltrating macrophages (Fig. 4a, b). These were observed in clusters within the endomysium and perimysium adjacent to regenerating myofibers and retained prominent F-PMO signal after 7 days, at which time, it was largely undetectable in myonuclei (Fig. 4b, Supplementary Fig. 5a). PMO was also seen in approximately half of resident macrophages diffusely distributed throughout non-inflamed regions of *mdx* and WT muscle but remained undetectable in myonuclei in these situations (Fig. 4c, d). Together, these observations suggest that this class of inflammatory cell is strongly predisposed to PMO uptake, perhaps through phagocytic mechanisms. The compact distribution of PMO within lesions strongly suggests that inflammatory exudation from the blood is responsible for PMO accumulation at specific sites within the muscle where it is retained long after the agent has been cleared from the systemic circulation^27^.

### PMO distribution parallels localization of dystrophin expression

**Fig. 5:**
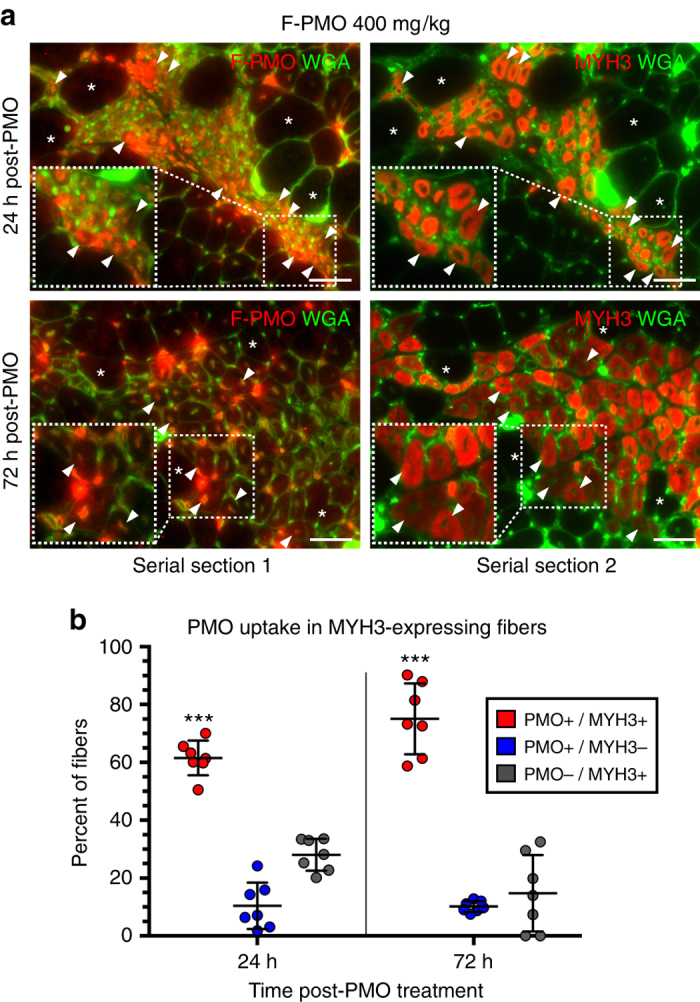
PMO uptake prevalent among actively regenerating myofibers expressing embryonic myosin heavy chain. **a** PMO localization and uptake specific to regenerating myofiber clusters expressing MYH3, after systemic F-PMO delivery in 4 week *mdx* mice (400 mg/kg, *n* = 3). Co-localization of PMO and MYH3^+ve^ myofibers examined in gastrocnemius, quadriceps, and triceps at 24 and 72 h post-PMO delivery. *Asterisks* denote fiber identity between serial cross-sections; *arrowheads* denote examples of MYH3^+ve^ myofibers with nuclear PMO. *Dashed boxes* show magnified inlay; *scale bars* represent 50 μm. **b** Quantification of PMO localization in MYH3^+ve^ myofibers shown as percent of labeled fibers. Statistical analysis performed by Mann–Whitney nonparametric test; ***p < 0.001. Data represented as scatter plot with SD

Our previous investigations of *mdx* muscle had found no correlation between mature fast or slow-twitch fiber types and the level of dystrophin restoration^25^, while acute cardiotoxin-induced muscle injury showed facilitated entry of PMO into regenerating fibers expressing embryonic myosin heavy chain (MYH3)—in both C57Bl/6 wild-type and *mdx52* dystrophin-null mice^29^. Here, we detected PMO for some 7 days after systemic administration where it was observed diffusely localized within interstitial spaces at scattered sites of inflammation and regeneration (Supplementary Fig. 2d), and was associated both with small-diameter, centrally nucleated, MYH3^+ve^ myofibers and mononucleated interstitial cells (Fig. 5a, b, Supplementary Fig. 5a, b). Fibers that were MYH3^+ve^ but lacked detectible PMO fell into two classes: those that did not contain a central nucleus within that given cross-section and those of larger diameter, suggesting that they had fully regenerated, but not fully matured, prior to PMO administration (Fig. 5b). Our general conclusion that PMO directly entered mononucleated cells, but not mature, multinucleated myofibers is subject to one exception; myonuclei of some spindle fibers located within regenerating lesions contained PMO but were not BrdU-positive. This was observed in spindle fibers residing in pathological lesions (Supplementary Fig. 5c, d). The thick capsule surrounding spindle fiber bundles does not commend them as obvious candidates for PMO penetration, thus we must look to some other quality that makes them uniquely susceptible to PMO uptake.

**Fig. 6:**
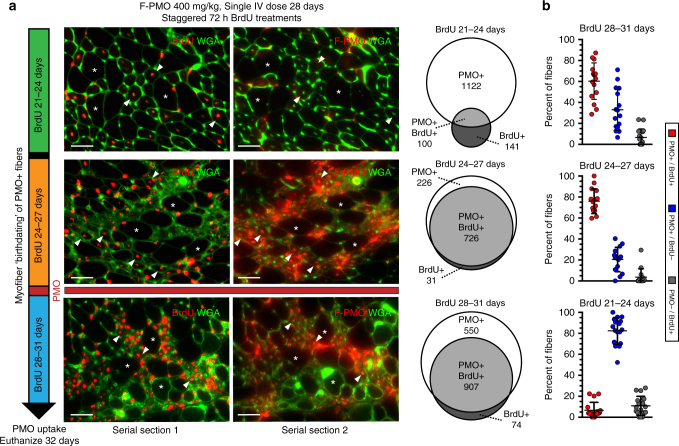
Robust PMO uptake in actively regenerating myofibers concurrent with systemic PMO delivery. **a** Myonuclear PMO uptake after single IV dose of F-PMO (400 mg/kg, *n* = 3) in 4 week *mdx* mice treated with staggered pulses of BrdU (refer to Fig. 1c “PMO uptake”). Note, *asterisks* denote fiber identity between serial cross-sections and *arrowheads* denote examples of centrally-nucleated BrdU^+ve^/PMO^+ve^ myofibers. Venn diagrams demonstrate the overlap between F-PMO localization (PMO^+ve^) and myofiber regeneration (BrdU^+ve^). *Scale bars* represent 50 μm. **b** Quantification of PMO^+ve^/BrdU^+ve^, PMO^+ve^/BrdU^−ve^, and PMO^−ve^/BrdU^+ve^ myofibers shown as percent of labeled fibers per BrdU cohort (*n* = 3 mice per cohort). Statistical analysis performed by Mann–Whitney nonparametric test; ***p < 0.001, **p < 0.01, *p < 0.05. Data represented as scatter plot with SD

Direct observation of PMO uptake was conducted on a second, parallel BrdU timing assay, employing F-PMO to permit precise histological localization. F-PMO (400 mg/kg) was injected into 28-day-old *mdx* mice in conjunction with 3-day BrdU treatments staggered from days 21–24, 24–27, and 28–31, with euthanasia on day 32 for immunofluorescent detection of residual F-PMO (Fig. 1b). Incompatibility of the immunostaining procedures for BrdU and PMO detection prevented direct co-localization and required comparison between serial cross-sections. Within regions of robust PMO localization of entire cross-sections of the gastrocnemius, triceps, and quadriceps, we assessed BrdU and PMO incorporation within individual fibers that could be tracked between adjacent serial sections. PMO and BrdU co-localized within nuclei exclusively within areas of regeneration and inflammation. Small-diameter, PMO/BrdU co-labeled myonuclei predominated in the two BrdU treatment cohorts adjacent to PMO delivery: ~76% in the 24–27 day BrdU cohort, and ~60% of fibers in the 28–31 day BrdU cohort (Fig. 6a, b). In contrast, in the 21–24 day BrdU cohort only 7% of fibers in PMO-enriched regions were PMO^+ve^/BrdU^+ve^, indicating poor PMO entry into fully formed myofibers (Fig. 6a, b).

The observation of PMO^+ve^/BrdU^+ve^ myonuclei in the 28–31-day treatment cohort indicates that some cells had undergone proliferation after exposure to PMO; this is intriguing in light of the short, <1 h half-life of PMO in the serum^25, 27, 28^, since it suggests that PMO is available for entry into regenerating myotubes for considerably longer than it is detectable in the serum. It is also possible that PMO had entered those myoblasts prior to the last cell cycle before terminal differentiation. These are not exclusive alternatives but the fact that residual PMO levels remained visually detectible within muscle lesions over this period inclines us toward the former explanation.

### PMO enters muscle precursor cells in dystrophic lesions

**Fig. 7:**
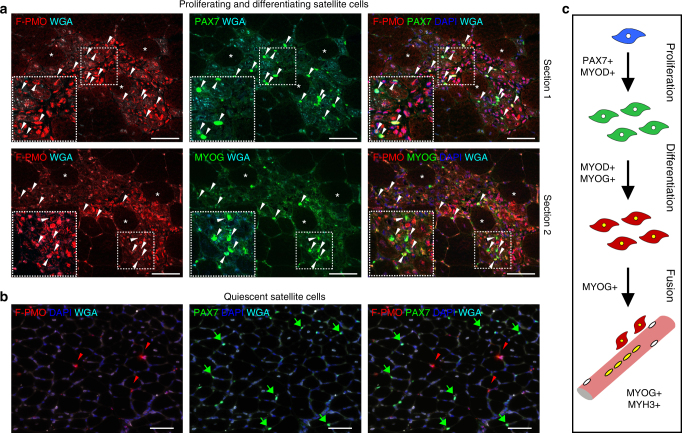
PMO infiltration of proliferating and differentiating myoblasts facilitates entry into actively regenerating fibers. **a** PMO uptake in proliferating (PAX7^+ve^, *top panel*) or differentiating (MYOG^+ve^, *bottom panel*) myoblasts within regenerating foci. *mdx* mice treated at 4 weeks with a single dose of F-PMO (400 mg/kg, *n* = 3) and euthanized 24 h after PMO delivery. *Asterisks* denote fiber identity between serial cross-sections; arrowheads denote PAX7^+ve^ or MYOG^+ve^ (*green*) cells co-labeled for PMO (*red*). Myoblasts void of PMO remain unmarked for comparison. Images acquired by confocal; *dashed boxes* show magnified inlay and *scale bars* represent 50 μm. **b** PAX7^+ve^ satellite cells residing in non-regenerating muscle regions do not co-localize with PMO^+ve^ nuclei. Arrowheads mark PMO^+ve^ nuclei. Merged image shows PMO (*red*), PAX7 or MYOG (*green*), WGA (*cyan*), and DAPI (*blue*). Gastrocnemius shown; *scale bars* represent 50 μm. **c** Schematic representation of myoblast proliferation, differentiation, and fusion with corresponding gene expression

We observed high incidence of PMO in mononucleated cells within inflammatory lesions for several days following systemic delivery of the tagged-PMO (400 mg/kg, single dose), many of which co-stained with the macrophage marker F4/80. However, significant numbers of PMO^+ve^ nuclei co-stained for paired box 7 protein (PAX7) or myogenic factor 4 (MYOG) (Fig. 7a, Supplementary Fig. 6), markers of satellite cell status and terminal myoblast differentiation, respectively (Fig. 7c). Meanwhile, quiescent, PAX7-expressing satellite cells residing at sites remote from muscle lesions contained no detectable PMO (Fig. 7b). Together, these results suggest that the robust uptake of PMO by myogenic precursor cells occurs exclusively within regenerating lesions, perhaps a reflection of the membrane changes associated with differentiation and fusion of myogenic cells together with the high local concentrations of PMO maintained within inflammatory lesions. This implicates myogenic precursor cells as integral mediators of PMO uptake into myofibers undergoing active repair.

### PMO uptake is augmented during myoblast differentiation and fusion

**Fig. 8:**
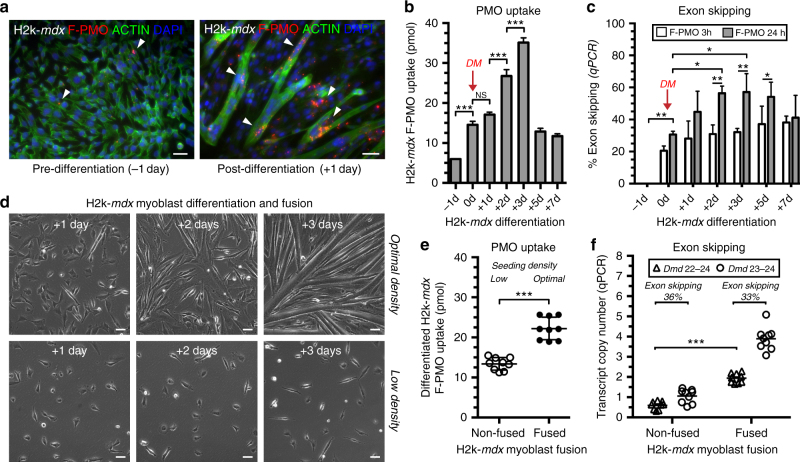
In vitro assessment of PMO uptake during myoblast differentiation and fusion. **a** F-PMO uptake in H2k-*mdx* myoblasts and fusing myotubes in vitro after a 24 h treatment with 100 μM F-PMO. F-PMO (*red*), actin (*green*), DAPI (*blue*). **b** Quantified F-PMO uptake (pmol) in H2k-*mdx* myoblasts at the designated time points pre-differentiation and post-differentiation after treatment with 100 μM F-PMO. **c** Exon skipping quantified by RT-qPCR as relative level of skipped vs. unskipped *Dmd* transcript 24 h following a 3 or 24 h F-PMO treatment (100 μM). Data represented as mean with SD. DM denotes change to differentiation media. **d** DIC imaging of H2k-*mdx* myoblasts at time points after onset of differentiation when seeded at optimal density to promote efficient myoblast fusion, or low density to inhibit myoblast fusion. **e** Quantified F-PMO uptake (pmol) in H2k-*mdx* cells treated with F-PMO (100 μM, 24 h) 2 days post-differentiation when maintained at optimal or low density. **f** *Dmd* mRNA transcript copy number quantified by RT-qPCR for skipped and unskipped transcripts 24 h after F-PMO treatment (100 μM, 24 h) in response to myoblast differentiation and fusion. Data represented as bar graph or scatter plot with SD. Statistical analysis performed by one-way ANOVA (**b**, **c**) or Mann–Whitney nonparametric test (**e**, **f**), ***p < 0.001, **p < 0.01, *p < 0.05. *Scale bars* represent 50 μm

Further investigations of the relationship between myoblast differentiation and fusion on PMO uptake and exon skipping were conducted in vitro. H2k-*mdx* myoblasts were maintained in 100 μM F-PMO for 24 h periods during proliferation and over the course of differentiation. In conditions permissive of active SV40 large T antigen, these cells proliferate, but when moved to non-permissive conditions (37 °C without IFNγ), switch rapidly to differentiation^39^. To mimic in vivo conditions, we used PMO concentrations calculated to correspond to the systemic concentrations immediately after IV delivery, assuming that these would be close to equilibrium with those within the inflammatory microenvironment of regenerating muscle foci (Supplementary Fig. 7a). Immunostaining for F-PMO revealed no significant uptake by proliferating myoblasts; however, onset of differentiation elicited a rapid intracellular uptake of PMO over the first 3 days of differentiation (Fig. 8a, b), amounting to a greater than 6-fold increase between proliferating and 3-day-differentiated cells (Fig. 8b). The abrupt drop in intracellular PMO on days 4–5, we attribute largely to the artifacts of detachment and fragility of myotubes at this stage (Fig. 8b). Exon skipping efficiency, monitored by RT-qPCR across this period, peaked between 2–5 days post-differentiation at nearly 60% skipped vs. full-length (Fig. 8c). At full differentiation, we noted the proportion of skipped *Dmd* transcript rose with duration of PMO exposure (i.e., 24 h compared to 3 h) (Fig. 8c). Myogenic differentiation was confirmed by Western blot for embryonic myosin (MYH3), which appeared at +1 day and peaked at +3 day (Supplementary Fig. 7c). We also examined the effect of prolonged PMO exposure on actively fusing myotubes, a scenario paralleling that in regenerating lesions in vivo, treating myotubes 3 days after onset of differentiation for 72 h with 50–500 μM PMO. We found that prolonged PMO exposure masked any significant effect of PMO dosage on exon skipping in culture (Supplementary Fig. 7b), identifying exposure time as a major factor in efficiency of exon skipping.

To further investigate the individual impacts of myoblast differentiation and fusion on uptake of PMO, we took advantage of the density-dependence of fusion efficiency to modulate myoblast fusion without pharmacological interference. Seeding density for optimal fusion (26,000 cells/cm^2^) was reduced by 75% to 6500 cells/cm^2^, thus preventing fusion under differentiation conditions (Fig. 8d). Two days after switching to non-permissive conditions, which corresponds to the peak of myoblast fusion under our optimal seeding conditions, cultures were treated with F-PMO (100 μM, 24 h). Differentiation was monitored by measuring Desmin (*Des*) and embryonic myosin (*Myh3*) mRNA transcript levels (Supplementary Fig. 7d, e). In our low-density, differentiating, non-fused myoblasts, intracellular F-PMO rose by ~2-fold over proliferating, undifferentiated myoblasts (Fig. 8b, e), but further increased to ~4-fold in fusing myoblast cultures (Fig. 8e). Thus, myoblast differentiation and fusion each contribute individually to efficient PMO uptake. This supports the idea that PMO is delivered into fusing myotubes both by PMO-loaded differentiating myoblasts and during the process of fusion itself.

Assessment of exon skipping by quantifying copy number of *Dmd 22*–*24* skipped relative to non-skipped transcript showed the same percent exon skipping, but revealed a significant increase in overall *Dmd* transcript copy number accompanying active myoblast fusion (Fig. 8f). Thus, the higher concentrations of PMO in cultured myotubes does not increase the percentage of exon skipping but does boost overall dystrophin transcription rate thus generating a greater total yield of skipped transcript. We conclude that (1) myoblast differentiation is sufficient to promote enhanced PMO uptake independently of myotube formation, and (2) concurrent myoblast differentiation and fusion promote robust PMO uptake through fusion of PMO-loaded myogenic cells during formation and by the myofiber repair process itself.

### PMO uptake by macrophages provides sustained PMO release in culture

**Fig. 9:**
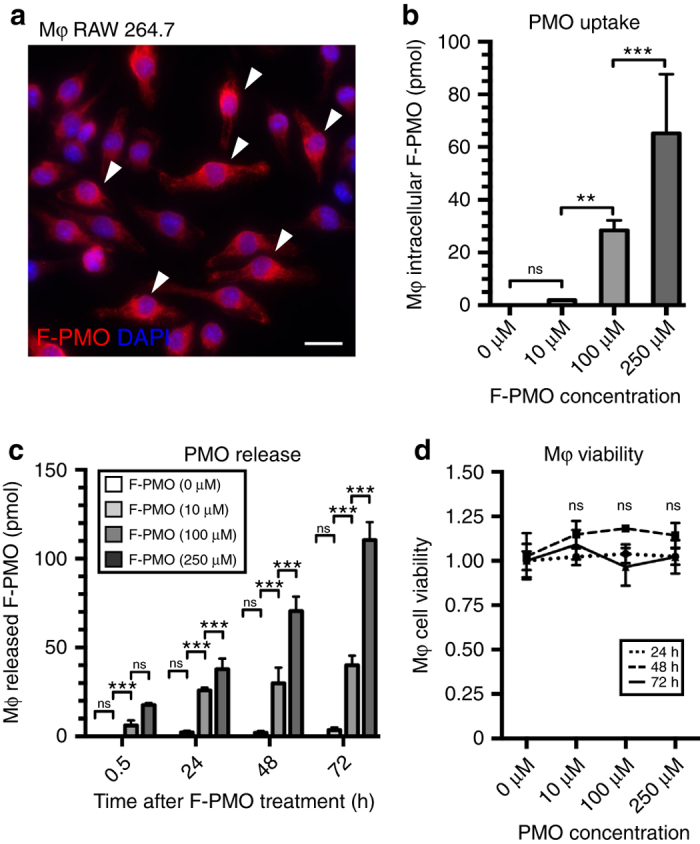
Investigation of PMO uptake and release in cultured macrophages. **a** F-PMO uptake in cultured RAW 264.7 macrophages (Mφ) after 24 h treatment with 100 μM F-PMO. F-PMO (red), DAPI (blue). *Arrowheads* mark PMO^+ve^ macrophages. *Scale bar* represents 20 μm. **b** Quantified F-PMO uptake (pmol) in cultured macrophages at designated time points after 24 h treatment of 10, 100, and 250 μM F-PMO. **c** Release of F-PMO (pmol) from cultured macrophages quantified at 0.5, 24, 48, and 72 h after 24 h F-PMO treatment (0, 10, 100, or 250 μM). **d** Normalized CCK-8 viability assay for cultured macrophages 24, 48, and 72 h, after 24 h PMO treatment. Statistical analysis performed by one-way ANOVA, ***p < 0.001, **p < 0.01, *p < 0.05

Much of the in vivo PMO accumulation in inflammatory foci is located within infiltrating macrophages, identifying them as a potential PMO repository within dystrophic lesions. When tested in vitro, RAW 264.7 macrophages treated for 24 h with 10–250 μM F-PMO showed robust uptake (Fig. 9a). Concentrations of 100 or 250 μM F-PMO produced a total intracellular F-PMO content of ~28 or ~65 pmol per cell pellet, respectively, even after the 3 days of proliferation in the absence of F-PMO (Fig. 9b). Cultured macrophages (3 × 10^6^), each with an approximate volume of 10^3^ µl, thus contained an estimated intracellular store of 8.6 and 19.8 μM, respectively (Supplementary Fig. 8a).

Next, we sought to test our hypothesis that release of PMO from macrophages may allow enhanced and prolonged PMO delivery to differentiating myoblasts in vivo. Macrophages were treated for 24 h with F-PMO (0, 10, 100, and 250 μM) then moved to fresh medium and aliquots were taken at 30 min, 24 h, 48 h, and 72 h to monitor the progressive accumulation of F-PMO released into the medium. Over 72 h, macrophages treated with an initial concentration of 100 or 250 μM F-PMO, released a total of 40 and 111 pmol F-PMO per culture well, corresponding to concentrations of 32 and 88 nM F-PMO, respectively, into the culture media (Fig. 9c, Supplementary Fig. 8b). Cell viability assay, by CCK-8 and trypan blue exclusion, 24, 48, and 72 h after removal of PMO, showed no evidence of accompanying cell death (Fig. 9d). We conclude that macrophages take up significant quantities of PMO, and subsequently release some 60% of their initial content over 72 h (Fig. 9b, c). This accords well with the idea that macrophages generate a reservoir of PMO at sites of muscle regeneration, which may subsequently be released intact in the vicinity of regenerating myotubes, thus extending the period of exposure of regenerating myofibers to PMO, long after it has disappeared from the serum.

### Repeat PMO delivery targets regenerating foci to increase dystrophin

**Fig. 10:**
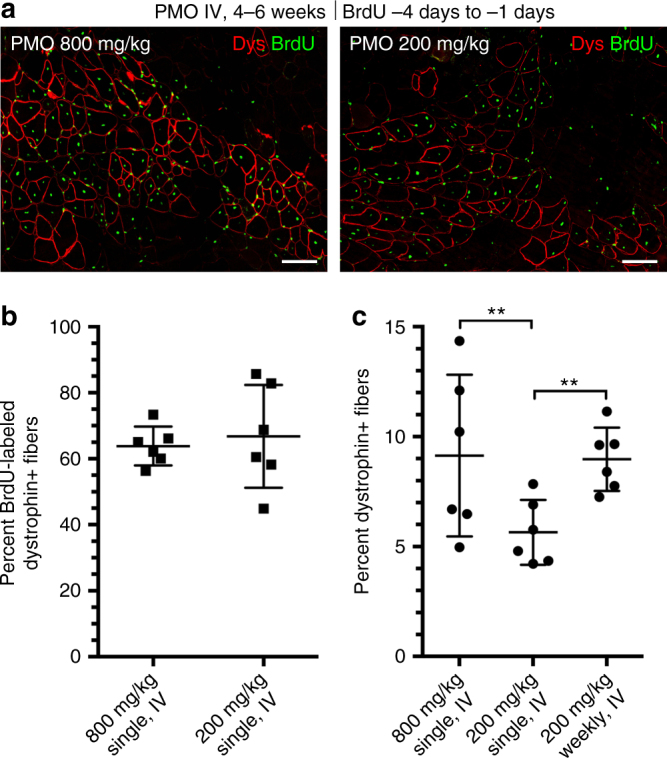
Role of myofiber repair in productive exon skipping consistent between low and high systemic PMO doses. **a** Dystrophin restoration and BrdU co-localization observed in 4–6 week *mdx* mice (i.e., triceps, gastrocnemius) after single, systemic dose of PMO (200 or 800 mg/kg, *n* = 3). BrdU administered for 72 h prior to IV PMO delivery. Dystrophin (Dys, *red*) and BrdU^+ve^ myonuclei (*green*) 14 days after PMO. *Scale bars* represent 100 μm. **b** Quantification of dystrophin and BrdU co-localization after 200 or 800 mg/kg PMO shown as percent of labeled fibers. **c** Quantification of dystrophin-expressing fibers after a single dose of 200 or 800 mg/kg or 4 weekly 200 mg/kg doses shown as percent of total fibers per cross-section. Data represented as scatter plot with SD. Statistical analysis performed by Mann–Whitney nonparametric test; ***p < 0.001, **p < 0.01, *p < 0.05

Our protocol of a single, high dose of PMO was designed specifically to elucidate the relationship between PMO entry and muscle pathology, but current PMO clinical studies employ repeated low-dose regimens. A conservative dose-by-factor approach to between-species translation^40^ equates a 20 mg/kg dose in humans to ~200 mg/kg in mice. Systemic injection of low-dose PMO (200 mg/kg) into 4–6-week-old *mdx* mice, both as a single dose and as four doses given at weekly intervals, was compared to a single high-dose (800 mg/kg) treatment. For both single-dose regimens, BrdU was given during the 3 days prior to PMO administration. This resulted in the same frequencies of dystrophin and BrdU co-labeling as in previous experiments, with some 60–65% of dystrophin-expressing fibers containing BrdU-labeled central nuclei, indicative of active regeneration prior to PMO delivery (Fig. 10a, b).

The single low-dose PMO administration produced significantly fewer dystrophin^+ve^ fibers (5.6%) than four weekly low-dose injections (8.9%), or the single high-dose PMO administration (9.1%) (Fig. 10c). Intriguingly, the effects of four weekly doses were not additive, resulting in only a doubling, rather than the expected quadrupling, of the number of dystrophin-expressing fibers, suggesting either turnover of those fibers or of the protein and/or a fluctuating rate of regeneration over the extended time frame. We conclude that the extent of muscle regeneration and associated inflammation at the time of systemic PMO delivery dictate both drug delivery and pharmacodynamics within individual muscles, and thus have direct implications for current PMO clinical trials where increasing both dose and frequency of delivery could have profound benefits.

## Discussion

A plausible antisense therapy for DMD must deliver functionally effective amounts of the therapeutic agent to a substantial proportion of myonuclei within large muscle groups distributed throughout the body, a target that can be reached comprehensively only by way of the blood vascular system. Currently the mechanisms regulating transfer of morpholino antisense from blood into cells, and the factors influencing their access to and interaction with the nascent dystrophin transcript are poorly understood. Furthermore, up to now, the basis of the pattern of dystrophin expression achieved through intravenous delivery of PMO in both preclinical and clinical investigations has defied elucidation^25^. For human exon skipping trials with ethical limitations on patient biopsy sampling, the resulting sparse Poisson distribution of the data has complicated interpretation^24^.

It has long been clear that effective PMO-induced exon skipping is dependent on some pathological aspect of the genetic defect and has been associated with pathological processes^29^; however, the precise cellular mechanisms have not previously been definitively identified and the notion that PMO entry is associated with a generalized “leakiness” of the dystrophic muscle fiber membrane is widely promulgated. Here, we have focused on the cellular and pathological mechanisms that impinge on delivery of PMO antisense into muscle fibers, which has been shown to correlate with the intensity of muscle pathology and to localize to lesions elicited by myotoxin injection^29^. Here, we further clarify this association by tying the level of exon skipping induced by systemically administered PMO to coincident myofiber regeneration and inflammatory exudation; two temporally linked but mechanistically separable features of muscular dystrophy pathology.

Gross pharmacokinetic analysis paints a picture in which ~90% of systemically delivered PMO is eliminated from the blood within the first hour, reaching baseline serum levels within 3 h and leaving barely detectable traces within muscle tissue^25–28^. This underlies a widely accepted view that entry into muscle fibers is limited to those that were in a susceptible state during this short period of systemic availability. Our analysis of PMO uptake at the cellular level transforms this model. First, it identifies the route of PMO into muscle fibers, whereby myogenic precursors mediate direct delivery into regenerating myofibers. Second, it implicates the inflammatory response, and macrophages in particular, as drug repositories that maintain PMO availability to myogenic cells for several days after its disappearance from the blood.

Our evidence identifies myogenic precursors as vehicles of direct delivery of PMO into regenerating myofibers. Shortly after administration F-PMO is observed in regenerating lesions within active (PAX7^+ve^) and differentiating (MYOG^+ve^) satellite cells, and later, within adjacent immature myotubes. It is also possible that entry occurs directly during the myoblast fusion process. Such a succession suggests a mechanism whereby cells lying in inflammatory and regenerating foci are readily penetrated by PMO, and cells committed to myogenesis mediate PMO entry into myofibers during the process of repair. This is in line with our observations in tissue culture where PMO entry was only marginally detectable in proliferating myoblasts, but became readily measurable during differentiation and yet more so, during fusion.

The overall strength of our case for the central role of myogenic cells in vivo comes from the use of “BrdU birthdating” of muscle fiber nuclei. This provides a common point of reference between penetration of F-PMO, occurrence of exon skipping, and production of dystrophin protein. Thus, the most conspicuous F-PMO penetration of differentiating myogenic cells occurred in those lesions that had undertaken the bulk of their proliferation during the previous 3 days. It was almost exclusively these same BrdU^+ve^ cells that subsequently gave rise to dystrophin-expressing muscle fibers, while the myogenic cells that had been proliferating more than 4 days earlier yielded only a minority of fibers that were penetrated by F-PMO and went on to produce dystrophin. Moreover, the sustained persistence of PMO in dystrophic lesions accounts for the unexpected finding of double-labeled dystrophin^+ve^/BrdU^+ve^ cells when BrdU was provided after the administration of PMO.

Our observations also identify inflammatory macrophages as a reservoir for prolonged availability of PMO to regenerating muscle fibers. Direct visual tracking of intravenously injected F-PMO revealed its persistence in localized patches, corresponding to areas of muscle damage, regeneration, and inflammation, for several days after its disappearance from the blood. Within these foci, diffuse PMO signal was visualized within interstitial spaces; however, the most robust signals were intracellular, within the myonuclei of small-diameter, newly formed, immature myofibers, and also within infiltrating inflammatory cells, a large proportion of which were macrophages.

Productive entry of PMO into muscle requires that the drug escape from the microvasculature into the interstitial space of inflammatory lesions generated by muscle fiber necrosis, putting it in intimate contact with susceptible cells of regenerating muscle. However, entry of PMO by equilibration across the inflamed microvascular bed should also predispose toward its loss in concert with the rapid drop in serum concentration, whereas we observed persistence of PMO for several days in inflamed sites. For such sustained PMO retention, the macrophage population is a clear suspect. Visually these cells form the main repository of F-PMO in these lesions in vivo. Furthermore, in culture, macrophages show avid PMO uptake and subsequent release of more than half of their PMO content over the following several days. Thus, they are capable of fulfilling a role as the persisting local source of PMO not in simple equilibrium with the serum, thereby acting to extend the duration of local drug availability. The question of whether all of the PMO is acquired while the macrophages reside within inflamed sites or whether some is actually phagocytosed in the blood and carried into the lesions is also of interest and suggests strategies for improving the efficiency of PMO delivery. Certainly, the chemical stability of PMO^41, 42^ would fit well with a schema whereby macrophages act as a buffering reservoir. Whether the release from macrophages is direct or via exosomes is also a topic of our future exploration. Likewise, the well-attested influence of macrophages on myogenic cell function via an array of cytokine interactions^43–50^ prioritizes examination of their role together with other components of the inflammatory environment^51–53^. Although intramuscular injection of PMO was found to induce strong levels of exon skipping^11, 12, 19^, this route of administration directly introduces local high concentrations of PMO into the interstitium rendering the exudative inflammatory component of our mechanism largely redundant; further, it adds mechanical damage and hydraulic expansion of the endomysial space. Together, this may account for the more impressive results seen in the first clinical trials that utilized intramuscular delivery^54^ than in subsequent trials involving systemic delivery^20^. An understanding of the dynamics of this system is clearly important to any attempt to optimize PMO-induced exon skipping in muscle.

We postulate that inflammatory foci act as local drug reservoirs for several days after the PMO had been eliminated from the circulation. Within these foci, PMO would have unimpeded access to the newly forming myofibers via differentiating and fusing myogenic precursors. Even at the high PMO doses we employed, we saw no evidence of direct entry of PMO into structurally intact extrafusal muscle fibers, nor did we observe PMO entry into quiescent satellite cells remote from regenerating muscle lesions. Thus, the nearly one-to-one relationship of dystrophin restoration in fibers labeled with BrdU during the 3 days prior to, or coincident with PMO delivery, suggests that productive PMO entry into dystrophic muscle fibers occurs via the regenerative process and argues against significant involvement of other mechanisms.

The above picture does not fit well with recent reports of successful exon skipping in the mdx mouse with chronic delivery of much lower doses of PMO than we have used^55^. In one case this was boosted by co-administration of PMO with hexoses, suggesting that cellular uptake of PMO occurs via an energy-dependent mechanism^56^. The main ATP requiring processes in our model are the proliferation and fusion of myoblasts and the phagocytic activity of macrophages, which are both difficult to reconcile with acute changes in ATP levels. How these would fit with our findings remains unclear but should be readily susceptible to investigation within our experimental model.

Our revised view of the mechanisms underlying productive PMO entry into muscle has direct implications for multi-exon skipping strategies for DMD^57^. Multiple skipping requires targeting of all components of an entire antisense cocktail to the same single nascent dystrophin transcript within a substantial proportion of myonuclei within individual myofibers; such a scenario is difficult to reconcile with a mechanism of low frequency, random, independent entry of each antisense component into the musculature. Our identification of a mechanism that would concentrate entry of all of the components of a PMO cocktail synchronously into a localized, punctate site via the repair process, provides a plausible rationale for this approach in DMD.

## Methods

### Animal studies

All animal procedures were thoroughly reviewed and given explicit prior approval by the Institutional Animal Care and Use Committee of Children’s National Health System (CNHS) in Washington DC. The C57Bl/10ScSn-*mdx*/J mouse model of DMD (*mdx*) was utilized for all experiments and harbors a nonsense point mutation in exon 23 of the dystrophin gene, thereby abolishing dystrophin protein expression^58^. The C57Bl/10ScSnJ (Bl10 or wild-type) mouse was used as the control where indicated. *mdx* and Bl10 mice ages 3 weeks to 4 months old were used in the various studies. Mouse strains were initially obtained from the Jackson Laboratory (Bar Harbor, ME). Mice were housed and bred at the CNHS Research Animal Facility, provided food, water and enrichment ad libitum, and maintained under 12 h light/dark cycles.

### Tissue harvesting and cryosectioning

Mice were euthanized at designated time points following drug administration via CO_2_ inhalation and cervical dislocation. Muscle samples including tibialis anterior, extensor digitorum longus, quadriceps, gastrocnemius, triceps, biceps, diaphragm, and heart were surgically removed, mounted on cork with tragacanth gum, flash-frozen in liquid nitrogen-chilled isopentane and stored at −80 °C. For immunohistochemistry, tissues were sectioned at 8 μm on a Leica CM1900 cryostat and stored at −80 °C; serial sections were utilized where indicated due to incompatible immunohistochemistry techniques or reagents.

### PMO antisense sequences and delivery

PMO targeting the exon-intron boundary of exon 23 of the dystrophin pre-mRNA (+7–18; 5′-GGCCAAACCTCGGCTTACCTGAAAT-3′)^12, 35, 36^ were synthesized (GeneTools, OR). A modified PMO23 oligo conjugated to fluorescein (+7–18; 5′-GGCCAAACCTCGGCTTACCTGAAAT-3′-Fluorescein) was also used to track the delivery and localization of the drug. PMO was diluted in saline and warmed at 50 °C for 15 min prior to injection. Mice were anesthetized with 4% isofluorane and PMO was administered systemically via the retro-orbital sinus as a single 200–800 mg/kg dose (<300 μl total volume) using a 31-guage/0.3 mL insulin syringe. Saline was injected at equivalent volumes as a control. Mice were euthanized at the designated time points indicated throughout the study.

### BrdU administration

5′-bromo-2′-deoxyuridine or BrdU (Sigma-Aldrich, MO) was administered ad libitum in drinking water at a concentration of 0.8 mg/ml for a period of 24–72 h. BrdU was prepared in sterile water and kept protected from light during administration. For BrdU pulse-labeling studies, BrdU was administered from 21 to 24 days (72 h), 24 to 27 days (72 h), 28 to 31 days (72 h) in conjunction with a single high dose of PMO delivered on the morning of day 28 coincident with BrdU administration for the 28–31 days cohort.

### Immunohistochemistry

Cryosections were stained with anti-dystrophin (GTX15277, 1:100, GeneTex, CA), anti-fluorescein (A889, 1:200, Life Technologies, MA), anti-BrdU-biotin conjugate (B35138, 1:100, Life Technologies, CA), anti-laminin-α2 (4H8-2, 1:100, Enzo, NY), anti-laminin (L9393, 1:400, Sigma-Aldrich, MO), anti-embryonic myosin heavy chain (MYH3) (F1.652, 1:25, Developmental Studies Hybridoma Bank, University of Iowa), anti-PAX7 (PAX7 concentrate, 1:20, Developmental Studies Hybridoma Bank, University of Iowa), anti-myogenin (sc12732, 1:100, Santa Cruz, TX), and F4/80 (CI-A3-1, 1:40, AbD Serotec, NC). Sections were fixed in ice-cold acetone for 10 min, washed in PBS (0.1% tween-20), and blocked for 1 h in PBS supplemented with 20% goat serum (GeneTex, CA), 0.1% tween-20 (Sigma-Aldrich, MO), and 10 mg/ml BSA (Sigma-Aldrich, MO). Primary antibodies were incubated overnight at 4 °C in a humidified chamber. Sections were then washed and probed with the appropriate Alexa Fluor secondary antibody (Life Technologies, MA) at a dilution of 1:500 for 1 h at room temperature. Wheat germ agglutinin (WGA) conjugated to Alexa Fluor 488, 568, or 647 (Life Technologies, MA) was prepared as 1 mg/ml stock solutions and used at a 1:500 dilution in PBS. Sections were mounted with Prolong Gold Mounting Media (Life Technologies, MA) with DAPI for nuclear staining if necessary. To probe for the F-PMO, fixation was performed in 4% paraformaldehyde for 10 min at room temperature; secondary Alexa Fluor 568 was utilized to exclude low-level fluorescence emitted by the fluorescein tag. For BrdU immunostaining, sections were fixed in ice-cold acetone for 10 min, incubated in 2 N HCl at 37 °C for 30 min, and briefly neutralized with 0.15 M sodium tetraborate (Sigma-Aldrich, MO); sections were blocked and incubated in the appropriate antibodies as described above.

### Microscopy

Microscopy was performed using an Olympus BX61 VS120-S5 Virtual Slide Scanning System with UPlanSApo 40×/0.95 objective, Olympus XM10 monochrome camera, and Olympus VS-ASW FL 2.7 imaging software. Additionally, images were acquired using an Olympus FV1000 Confocal Microscope with UPlanFLN 40×/1.30 oil objective and Olympus FV-ASW version 4.2 imaging software. Live cell culture imaging was performed using an Olympus IX81 with LUCPlan FLN 20×/0.45 objective, Olympus XM10 monochrome camera and Olympus CellSens 1.13 software. Analysis and quantification were performed using Olympus CellSens 1.13, MetaMorph Premier 7.7.0.0, Adobe Photoshop CS6, and ImageJ software.

### PMO/dystrophin and BrdU co-localization quantification

Quantification of PMO/dystrophin and BrdU co-localization was performed on gastrocnemius, triceps, and quadriceps of *mdx* mice treated as described above. Regions were selected based primarily on clustered PMO at 4 days or dystrophin immunostaining at 14 days post PMO administration. Average fiber number per region was ~85 fibers for PMO-BrdU immunostained tissue and ~175 fibers per region for dystrophin-BrdU immunostained tissue. Regions of early regeneration and/or severe inflammation were not included in quantification if a discernable fiber membrane was lacking (i.e., diameter <10 μm) for a given PMO^+ve^ or BrdU^+ve^ nucleus. Gastrocnemius, triceps, and quadriceps were analyzed (*n* = 3 mice per treatment cohort). For PMO-BrdU quantification, serial cross-sections were utilized due to incompatibility of immunostaining procedures. Fibers were classified as PMO^+ve^/BrdU^+ve^, PMO^+ve^/BrdU^−ve^, and PMO^−ve^/BrdU^+ve^ or dystrophin^+ve^/BrdU^+ve^, dystrophin^+ve^/BrdU^−ve^, and Dys^−ve^/BrdU^+ve^. Percentages were calculated based on total PMO/dystrophin^+ve^ and BrdU^+ve^ fibers quantified per region per muscle.

### TaqMan RT-qPCR

Total RNA was extracted from muscle biopsies using TRIzol (Life Technologies, MA). Total RNA (400 ng) was reverse transcribed using the High Capacity cDNA Reverse Transcription Kit (Applied Biosystems, CA) following the manufacturer’s protocol with one modification; the random primer mix was replaced with forward (5′-CAGAATTCTGCCAATTCGTGAG-3′) and reverse (5′-TTCTTCAGCTTGTGTCATCC-3′) primers amplifying exons 20–26 (Integrated DNA Technologies, IA). cDNA (10 ng) was loaded for each triplicate reaction and analyzed using mouse-specific TaqMan probes (Life Technologies, MA) on the 7900HT Fast Real-Time PCR system. The TaqMan gene expression probe for the skipped product (Life Technologies Assay ID# AIOIXIL) was custom designed to amplify the splice junction at exon 22–24. For the non-skipped product, TaqMan gene expression probe (Life Technologies Assay ID# Mm0126935_m1) was used to amplify the region spanning exons 23–24. Absolute quantitation of muscle-specific gene expression was calculated using a standard curve derived from a known quantity of DNA plasmids. The skipped product fragment (exon 22–24) and the non-skipped product fragment (exon 23–24) were cloned separately into pMA-T vectors (Life Technologies, MA), purified and concentrated. Percentage exon skipping was calculated using the following equation: [Average of triplicate reactions of skipped DMD/(Average of triplicate reactions of skipped DMD + Average of triplicate reactions of non-skipped DMD)] × 100. *Dmd* skipped and unskipped transcript copy number was calculated based on a standard curve and represents transcript copy number per 10 ng extracted RNA. Additionally, *Des* expression assessed using TaqMan gene expression probe Mm00802455_m1; *Myh3* expression assessed using TaqMan gene expression probe Mm01332463_m1 (Life Technologies, MA); here, total RNA was reversed transcribed using a random primer mix according to the manufacturer’s protocol (Applied Biosystems, CA).

### Nested RT-PCR

Total RNA was isolated and reverse transcribed as described above. Primary PCR using 350 ng of cDNA was carried out using the Platinum TaqPCRx DNA Polymerase (Thermo Fisher, MA) with a hot start at 94 °C for 2 min, followed by 30 cycles of 95 °C (30 s), 55 °C (1 min), and 72 °C (2 min). Approximately 20 ng of the primary PCR products were re-amplified using the Platinum TaqPCRx DNA Polymerase (Thermo Fisher, MA) with forward (5′-CCCAGTCTACCACCCTATCAGAGC-3′) and reverse (5′-CAGCCATCCATTTCTGTAAGG-3′) primers amplifying exons 20–24. The secondary PCR was carried out with a hot start at 94 °C for 2 min, followed by 22 cycles of 95 °C (30 s), 55 °C (1 min), and 72 °C (2 min). Secondary PCR products were analyzed on a 2% agarose gel to identify skipped and unskipped dystrophin transcripts.

### Cell culture and PMO quantification

H2k-*mdx* myoblasts were maintained in DMEM, high glucose (4500 mg/L), pyruvate (100 mg/L) and GlutaMAX (Life Technologies, MA) supplemented with 20% heat inactivated FBS (Atlanta Biologicals, GA), 2% chick embryo extract (United States Biological) and 1% penicillin/streptomycin (100 U/mL) (Life Technologies, MA) on 0.4% gelatin-coated plates. Myoblasts were proliferated (5000 cells/cm^2^) at 33 °C with 10% CO_2_ and 20 ng/ml of IFNγ (R&D Systems, MN). For differentiation, myoblasts (26,000 cells/cm^2^) were plated onto 24-well 0.4% gelatin-coated plates; after 24 h the media was aspirated and replaced with differentiation media (DM) containing DMEM, high glucose (4500 mg/L), pyruvate (100 mg/L) and GlutaMAX supplemented with 5% horse serum and 1% penicillin/streptomycin (100 U/mL) (Life Technologies, MA). Cells were differentiated for up to 7 days at 37 °C with 5% CO_2_. To inhibit myotube formation, myoblasts were seeded at 25% of optimal density (6500 cells/cm^2^) and maintained under differentiation conditions. F-PMO was delivered to cultured cells at concentrations of 10–500 μM for 3–72 h at the designated time points to assess F-PMO uptake and exon skipping levels. Concentrations mimic the approximate PMO concentration achieved after systemic PMO delivery (i.e., 400 mg/kg yields an immediate systemic concentration ~500 μM, respectively, based on approximate blood volume, which drops to ~50 μM within 1 h). RAW 264.7 macrophages (ATCC, VA) were cultured in DMEM, high glucose (4500 mg/L), pyruvate (100 mg/L) and GlutaMAX supplemented with 10% heat inactivated FBS and 1% penicillin/streptomycin (100 U/mL) (Life Technologies, MA) at 37 °C with 5% CO_2_. RAW intracellular F-PMO concentrations were estimated based on cell density (3 × 10^6^ cells per well) and approximate cell volume (1000 μm^3^). For all assays, intracellular and released F-PMO was quantified by fluorescence intensity on a fluorescent plate reader. Exon skipping levels were evaluated from purified RNA extracted 24 h after PMO treatment during myoblast differentiation (change from growth media to DM noted as day 0). Western blot analysis was performed to confirm H2k cell differentiation during the PMO treatment time course; lysates were collected at the conclusion of each 24 h PMO treatment and subsequently run on NuPAGE Novex 3–8% tris-acetate protein gels (Thermo Fisher, MA) under denaturing conditions. Antibodies against MYH3 (F1.652, 1:1000, Developmental Studies Hybridoma Bank) and β-actin (sc-47778, Santa Cruz Biotechnology, TX) were utilized. Cell viability was determined using trypan blue or CCK-8 viability reagent (Sigma-Aldrich, MO) according to the manufacturer’s protocol.

### Statistical analyses

Statistical comparisons were performed by Mann–Whitney non-parametric rank sum test or one-way analysis of variance (ANOVA) test based on statistical assumptions and normality of data distributions. Data are typically reported as scatter plots ±SD. In all experiments, the differences were considered statistically significant when *p* < 0.05 and was reported as follows: ***p < 0.001, **p < 0.01, *p < 0.05.

### Data availability

The data that support the findings of this study are available from the corresponding author upon reasonable request.

## Electronic supplementary material

Supplementary Information(PDF 17217 kb)
