# Author Correction: Myoblasts and macrophages are required for therapeutic morpholino antisense oligonucleotide delivery to dystrophic muscle

**DOI:** 10.1038/s41467-017-02206-8

**Published:** 2018-01-15

**Authors:** James S. Novak, Marshall W. Hogarth, Jessica F. Boehler, Marie Nearing, Maria C. Vila, Raul Heredia, Alyson A. Fiorillo, Aiping Zhang, Yetrib Hathout, Eric P. Hoffman, Jyoti K. Jaiswal, Kanneboyina Nagaraju, Sebahattin Cirak, Terence A. Partridge

**Affiliations:** 1 Center for Genetic Medicine Research, Children’s Research Institute, Children’s National Health System, Washington, DC 20010 USA; 2 Institute for Biomedical Sciences, The George Washington University School of Medicine and Health Sciences, Washington, DC 20052 USA; 3 Department of Pediatrics, The George Washington University School of Medicine and Health Sciences, Washington, DC 20052 USA; 4 Department of Pharmaceutical Sciences, School of Pharmacy and Pharmaceutical Sciences, Binghamton University, Binghamton, NY 13902 USA; 5 Institute for Human Genetics, University Hospital Cologne, Cologne, 50923 Germany; 6 Department of Pediatrics, University Hospital Cologne, Cologne, 50923 Germany; 7 Center for Molecular Medicine, University of Cologne, Cologne, 50931 Germany

**Keywords:** Drug delivery, Macrophages, Neuromuscular disease, Skeletal muscle

*Nature Communications* **8**:941 10.1038/s41467-017-00924-7; Published online: 16 October 2017

In the original version of this Article, financial support was not fully acknowledged. The PDF and HTML versions of the Article have now been corrected to include support from the CRI Light Microscopy and Image Analysis Core.

Microscopic analysis for this study was conducted at the CRI Light Microscopy and Image Analysis Core, which is supported by CRI and the Intellectual and Developmental Disabilities Research Center Award (U54HD090257) through the National Institutes of Health NICHD.

