# Author Correction: Myoblasts and macrophages are required for therapeutic morpholino antisense oligonucleotide delivery to dystrophic muscle

**DOI:** 10.1038/s41467-018-03709-8

**Published:** 2018-03-23

**Authors:** James S. Novak, Marshall W. Hogarth, Jessica F. Boehler, Marie Nearing, Maria C. Vila, Raul Heredia, Alyson A. Fiorillo, Aiping Zhang, Yetrib Hathout, Eric P. Hoffman, Jyoti K. Jaiswal, Kanneboyina Nagaraju, Sebahattin Cirak, Terence A. Partridge

**Affiliations:** 1 Center for Genetic Medicine Research, Children’s Research Institute, Children’s National Health System, Washington, DC 20010 USA; 2 Institute for Biomedical Sciences, The George Washington University School of Medicine and Health Sciences, Washington, DC 20052 USA; 3 Department of Pediatrics, The George Washington University School of Medicine and Health Sciences, Washington, DC 20052 USA; 4 Department of Pharmaceutical Sciences, School of Pharmacy and Pharmaceutical Sciences, Binghamton University, Binghamton, NY 13902 USA; 5 Institute for Human Genetics, University Hospital Cologne, 50923 Cologne, Germany; 6 Department of Pediatrics, University Hospital Cologne, 50923 Cologne, Germany; 7 Center for Molecular Medicine, University of Cologne, 50931 Cologne, Germany

**Keywords:** Drug delivery, Macrophages, Neuromuscular disease, Skeletal muscle

Correction to: *Nature Communications* 10.1038/s41467-017-00924-7, published online 16 October 2017

The originally published version of this Article contained an error in Figure 6. In panel b, the top graph (BrdU 21–24d) and the bottom graph (BrdU 28–31d) were inadvertently swapped. This error has now been corrected in both the PDF and HTML versions of the Article.

