# Supplementary material for: Myoblasts and macrophages are required for therapeutic morpholino antisense oligonucleotide delivery to dystrophic muscle
Source: Nat Commun. 2017 Oct 16;8:941. doi: 10.1038/s41467-017-00924-7 (PMC5643396; doi:10.1038/s41467-017-00924-7)
Supplement: Supplementary file 1 — Supplementary Information(PDF 17217 kb) [file 41467_2017_924_MOESM1_ESM.pdf]

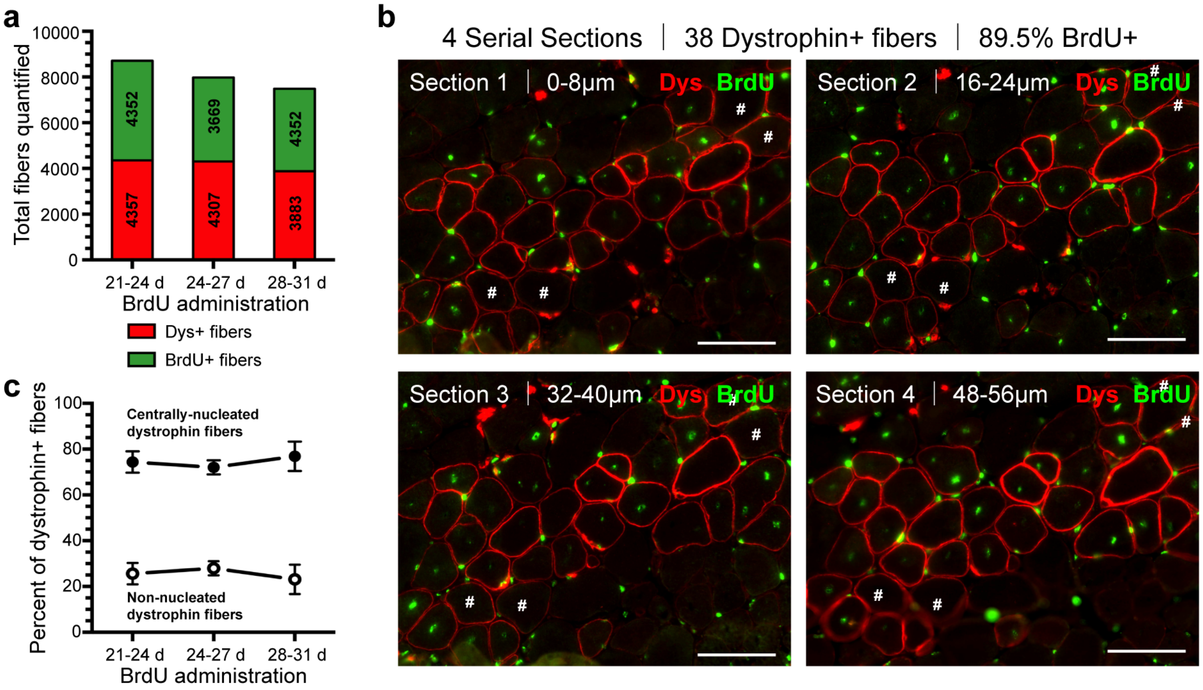

Supplementary Figure 1. BrdU labeling of regenerating myonuclei in dystrophic muscle. a. Total number of dystrophin^{+ve} and BrdU^{+ve} fibers quantified in Fig. 2 for each BrdU-treatment cohort. Total numbers of dystrophin and BrdU labeled fibers remains constant between the different BrdU cohorts. **b.** Serial cross-section analysis of dystrophin^{+ve}, BrdU^{+ve} myofiber clusters to determine frequency of central nuclei along the length of newly regenerated *mdx* myofibers. # denote myofibers where a BrdU^{+ve} central nuclei was not observed in any cross-section, in contrast to the majority of myofibers where BrdU^{+ve} central myonuclei were observed in at least 1 in 4 cross-sections analyzed. **c.** Quantification of dystrophin^{+ve} centrally-nucleated fibers (CNF) and dystrophin^{+ve} non-CNF fibers per muscle cross-section following PMO treatment (800 mg/kg, n=3 mice per cohort); at least 5 muscles were analyzed per cohort.

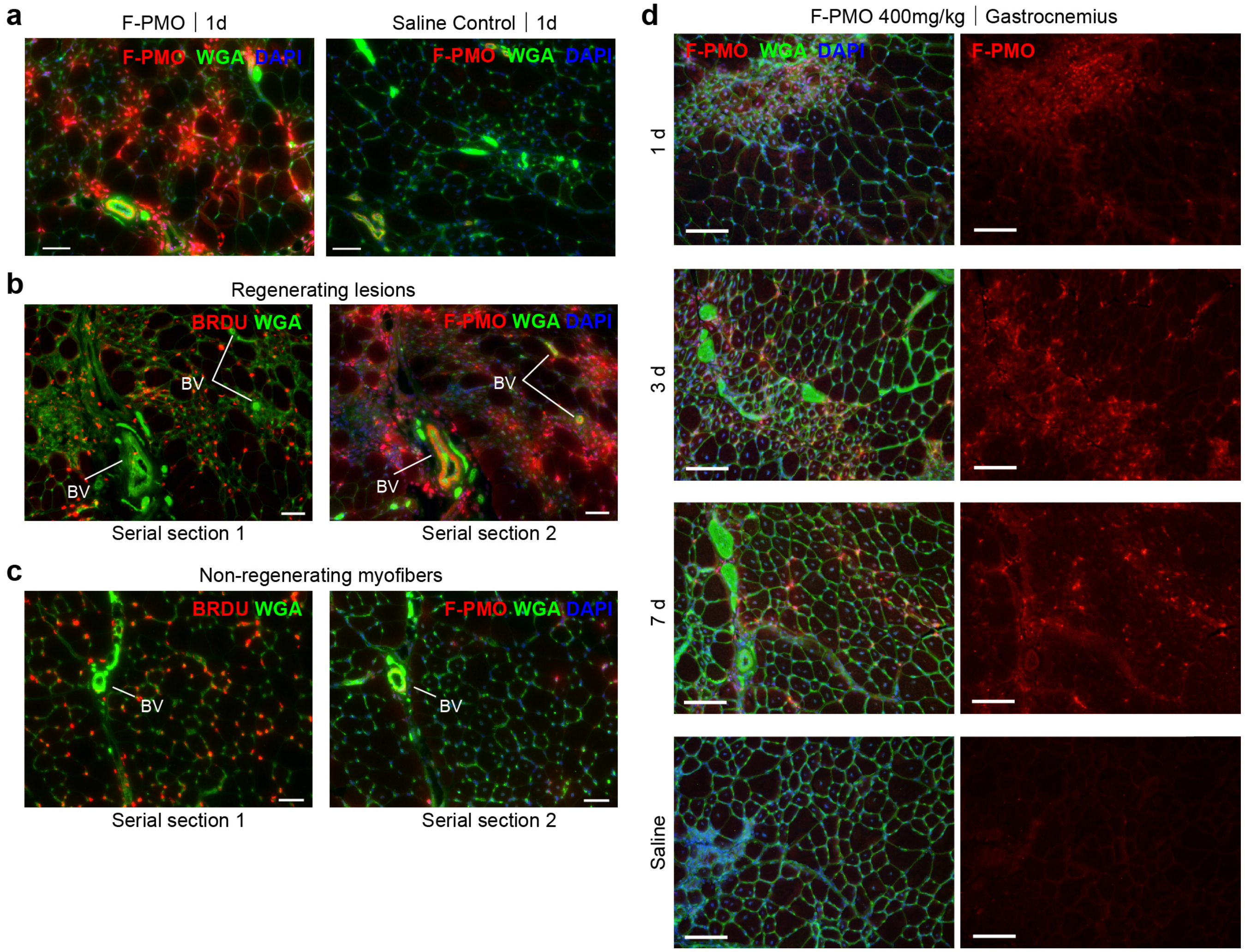

Supplementary Figure 2. Detection of F-PMO after systemic high-dose delivery by immunofluorescence. a. Myonuclear uptake of F-PMO 24 h after systemic delivery (400 mg/kg, n=3) in 4 wk *mdx* mice; gastrocnemius shown. F-PMO (red), WGA (green) and DAPI (blue). Saline-treated *mdx* muscle shown as control. **b, c.** PMO uptake in regenerating, BrdU^{+ve} myofibers and interstitial, mononuclear inflammatory cells (**b**); non-regenerating regions show no PMO uptake (**c**). BV, blood vessel. **d.** Myonuclear localization of PMO 1 d, 3 d, and 7 d following a single, systemic dose of F-PMO (400 mg/kg, n=1) in 5 week old *mdx* mice. PMO was primarily localized within the myonuclei of recently regenerated myofiber clusters (depicted as small-diameter, centrally-nucleated myofibers) in young *mdx* mice. PMO (red), WGA (green) and DAPI (blue). Scale bars represent 100 µm.

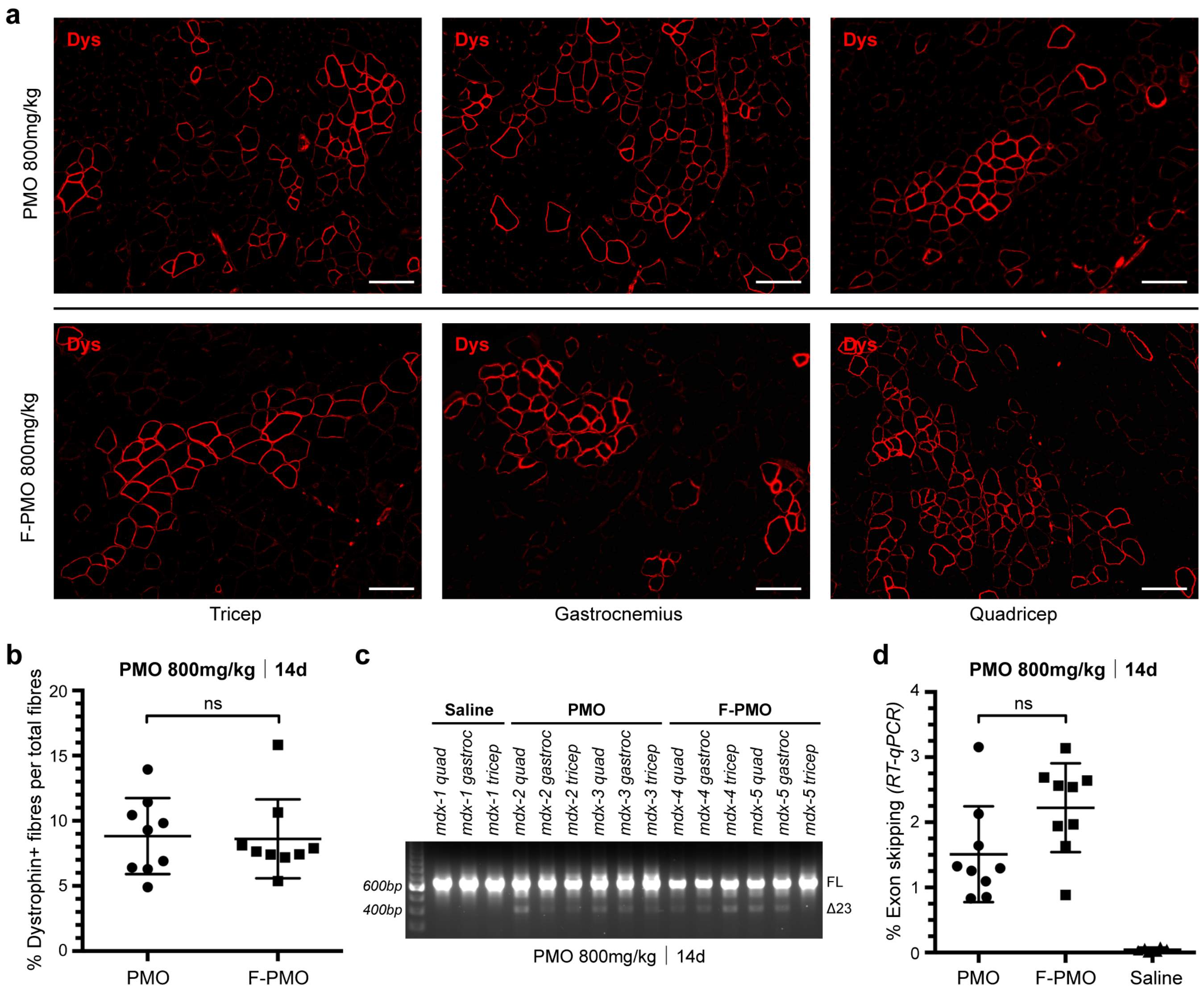

Supplementary Figure 3. Efficacy comparison of untagged-PMO and F-PMO in *mdx* mice.
a. Immunofluorescence of dystrophin expression after systemic delivery of 800 mg/kg PMO (top panel) or F-PMO (bottom panel) in *mdx* (n=3). Scale bars represent 100 µm. **b.** Percent dys^{+ve} fibers per total fibers per cross-section assessed 14 d after systemic delivery of 800 mg/kg PMO or F-PMO in *mdx* (n=3). Data correspond to muscles depicted in ***panel a*** and represented as mean ± SD. **c.** Relative levels of exon skipping assessed by nested RT-PCR 14 d after systemic delivery of 800 mg/kg PMO or F-PMO (n=3). Skipped *Dmd* PCR products corresponding to full-length *Dmd* transcript (FL), *Dmd* Δ23. **d.** Exon skipping quantified by RT-qPCR as relative level of skipped versus unskipped *Dmd* transcript 14 d after systemic delivery of 800 mg/kg PMO or F-PMO (n=3); data correspond to triceps, quadriceps, and gastrocnemius for each mouse and represented as mean ± SD. Statistical analysis performed by parametric t-test.

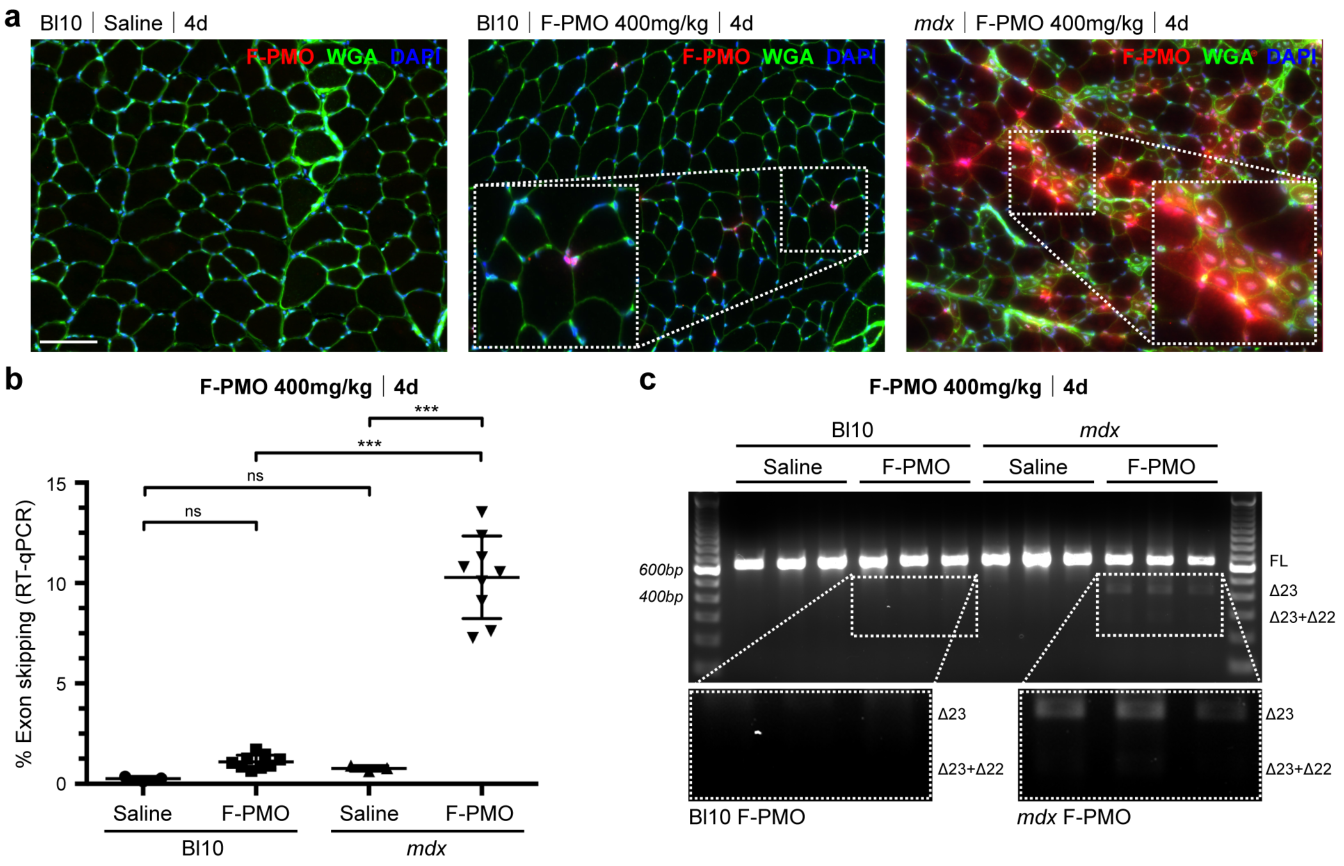

Supplementary Figure 4. Comparison of F-PMO uptake and efficacy in *mdx* and wild type muscle. **a.** Comparison of F-PMO uptake in *mdx* and wild type Bl10 muscle (quadriceps shown) revealed minimal interstitial accumulation of F-PMO in wild type muscle compared to *mdx (n=3).* Dashed boxes show magnified inlay. Scale bars represent 100 µm. **b.** Exon skipping quantified by RT-qPCR as relative level of skipped versus unskipped *Dmd* transcript 4 d following systemic F-PMO delivery (400 mg/kg) for Bl10 and *mdx* mice following administration of saline or F-PMO (400 mg/kg). n=3 mice per cohort; data corresponds to triceps, quadriceps, and gastrocnemius for each mouse and represented as mean ± SD. Statistical analysis performed by one-way ANOVA; *** < 0.001. **c.** Relative levels of exon skipping assessed by nested RT-PCR for Bl10 and *mdx* mice following administration of saline or F-PMO (400 mg/kg, n=3). Dashed boxes show magnified inlay. Skipped *Dmd* PCR products corresponding to full-length *Dmd* transcript (FL), *Dmd* Δ23, and *Dmd* Δ23+ Δ22 are shown. Skipped products only detectible for F-PMO treated *mdx* cohort.

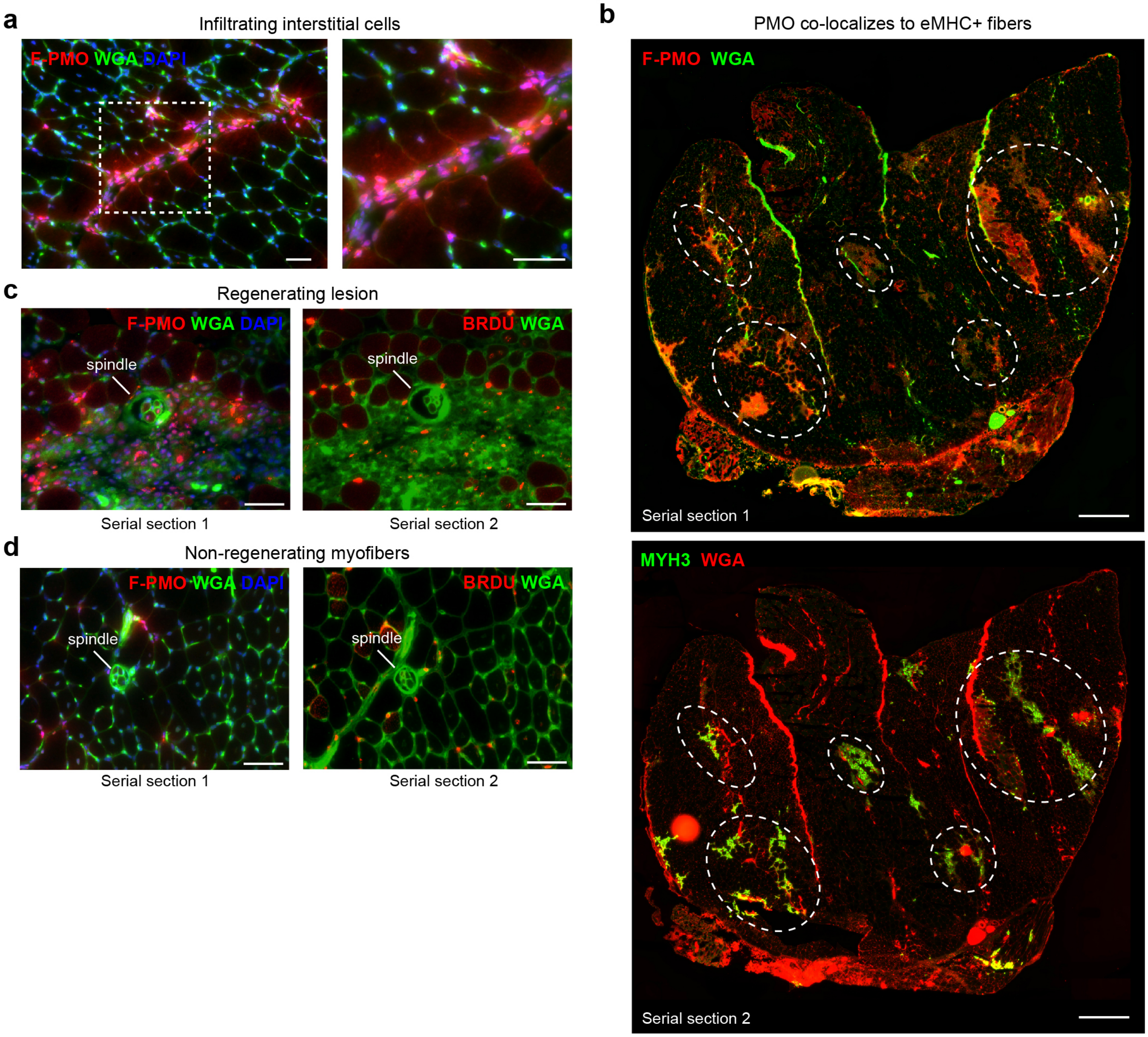

Supplementary Figure 5. Accumulation of PMO in regions of regeneration and inflammation in *mdx* mice. a. Robust PMO uptake within interstitial inflammatory cells 4 d following tagged-PMO delivery (400 mg/kg, n=3). PMO uptake not observed within adjacent non-regenerating fibers. Triceps shown. **b.** F-PMO uptake (top) and MYH3 expression (bottom) on adjacent whole cross-sections following injection of F-PMO (400 mg/kg, n=3). Tissue harvested 4 d after PMO. Scale bars represent 500 μm. **c, d.** PMO uptake in myonuclei of spindle fibers in proximity to areas of muscle regeneration (**c**); spindles within non-regenerating regions show no PMO uptake (**d**). *mdx* mice administered F-PMO (400 mg/kg) at 28 d and then subsequently dosed with BrdU from days 28-31 prior to tissue harvesting on day 32. Scale bars represent 50 μm.

a

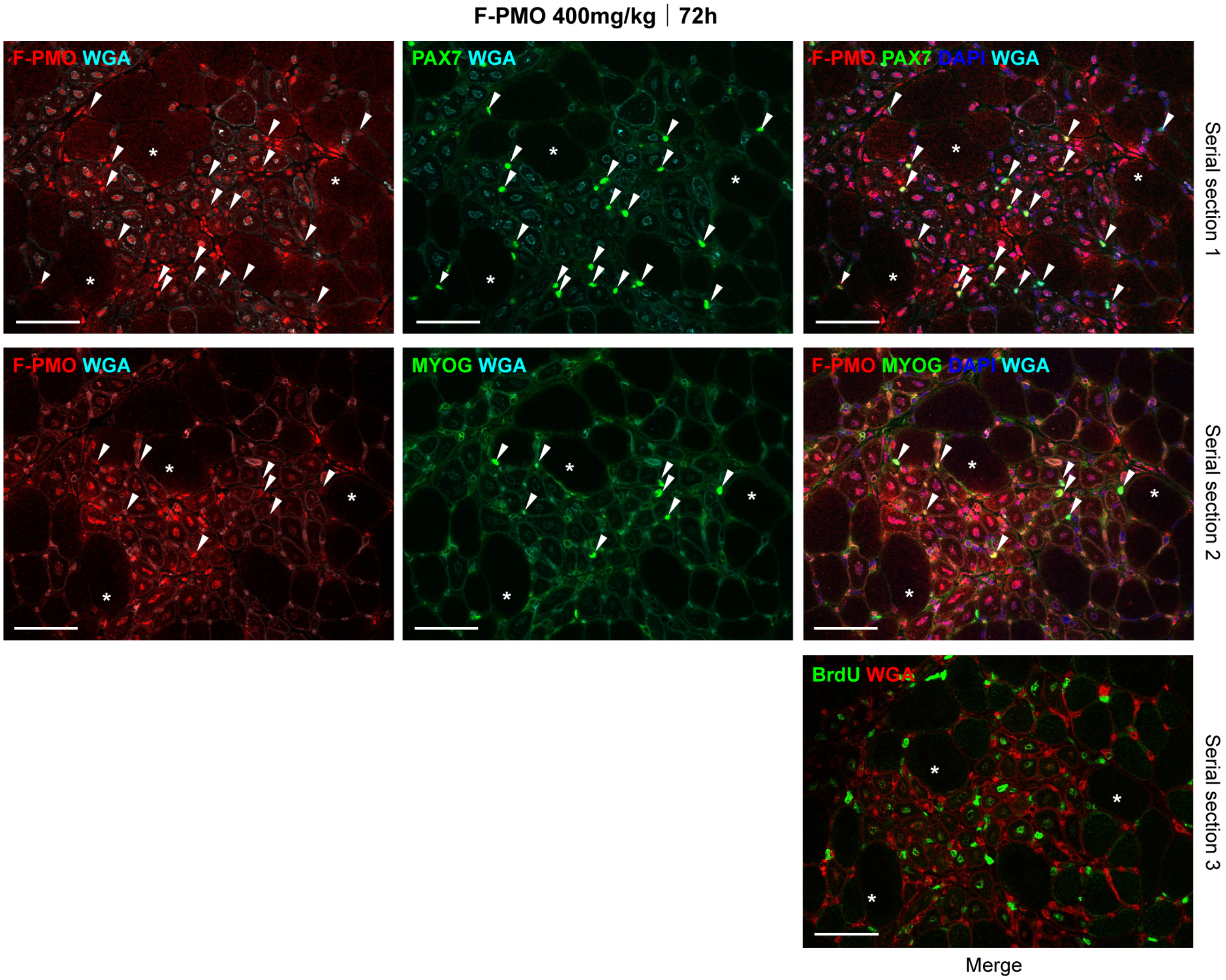

Supplementary Figure 6. PMO penetrates differentiating myoblasts within regenerating lesions. a. PMO infiltration of myoblasts 72 h following systemic delivery of F-PMO (400 mg/kg, n=3). BrdU co-administered during the 3 days prior to PMO delivery to assess level of regeneration. Serial section 1 (top) shows PMO uptake (red) relative to PAX7^{+ve} myoblasts (green). Serial section 2 (middle) shows PMO uptake (red) relative to MYOG^{+ve} myoblasts (green). Serial section 3 (bottom) corresponds to BrdU (green) co-stained with WGA (red). * denote fiber identity between serial cross-sections; arrowheads denote PAX7^{+ve} or MYOG^{+ve} (green) cells that co-label for PMO (red). Myoblasts void of PMO remain unmarked for comparison. Gastrocnemius shown; images acquired by confocal, Scale bars represent 50 μm.

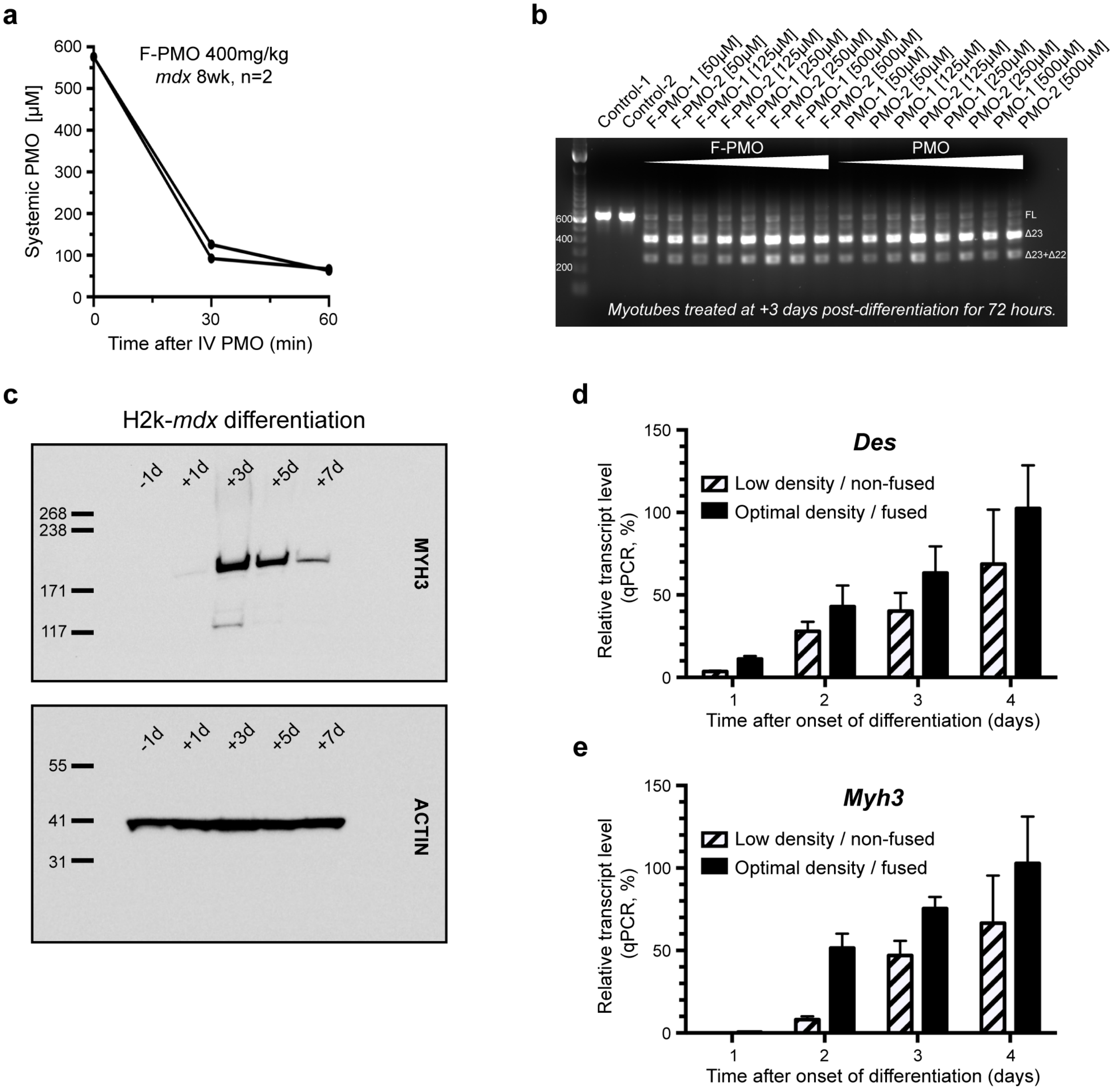

Supplementary Figure 7. Assessment of differentiating H2k-*mdx* myoblasts after PMO treatment in culture. a. Circulating PMO concentration after systemic delivery of 400 mg/kg F-PMO in 8 wk *mdx* mice (n=2). Blood collection taken at 30 and 60 min after dosing. Initial circulating PMO concentration estimated from dosage and estimated blood volume. **b.** Exon skipping assessed by nested RT-PCR for cultured differentiating/fusing H2k-*mdx* myoblasts. Cells were treated with F-PMO or PMO at 3 days post-differentiation at the concentrations indicated. Skipped *Dmd* PCR products corresponding to full-length *Dmd* transcript (FL), *Dmd* Δ23, and *Dmd* Δ23+Δ22 are shown. Conditions were tested in duplicate. Statistical analysis performed by one-way ANOVA; *** < 0.001, ** < 0.01, * < 0.05. **c.** Western blot analysis of cultured H2k-*mdx* myoblasts at staggered time points during differentiation/fusion corresponding to the start of each respective PMO treatment. MYH3 and ACTIN are shown. **d, e.** Relative mRNA transcript levels for desmin (*Des*) (**d**) and embryonic myosin heavy chain (*Myh3*) (**e**) quantified by RT-qPCR normalized to HRPT expression at time points during myoblast differentiation and fusion; conditions were tested in triplicate.

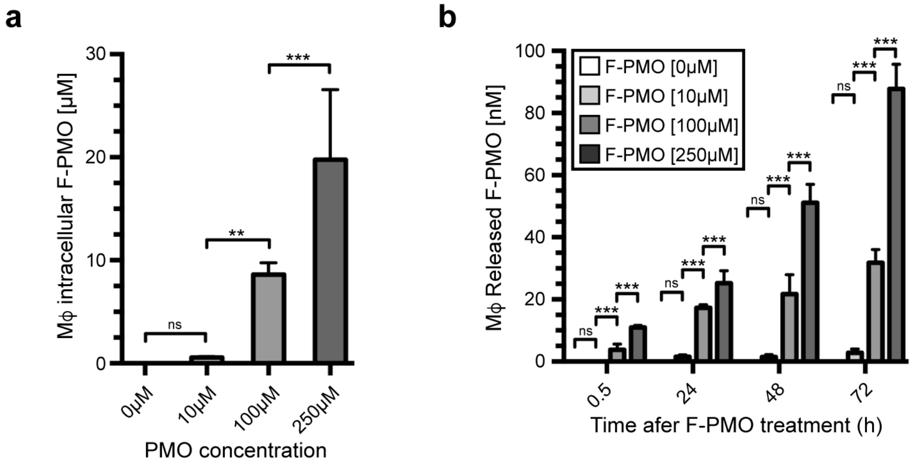

Supplementary Figure 8. Assessment of PMO uptake and release in cultured RAW macrophages. **a.** Quantified F-PMO uptake represented as intracellular molarity in RAW macrophages at the designated time points after a 24 h treatment of 10 μM, 100 μM, and 250 μM; lysates taken after treatment and normalized to 1 mg/ml in RIPA. **b.** Release of F-PMO (reported as [nM]) from RAW macrophages quantified at 0.5 h, 24 h, 48 h, and 72 h after a 24 h F-PMO treatment (0 μM, 10 μM, 100 μM, or 250 μM); molarity based on culture volumes per well. Conditions were tested in triplicate. Statistical analysis performed by one-way ANOVA; *** < 0.001, ** < 0.01, * < 0.05.
